# Photoresponsive Amide-Based Derivatives of Azobenzene-4,4′-Dicarboxylic Acid—Experimental and Theoretical Studies

**DOI:** 10.3390/ma14143995

**Published:** 2021-07-16

**Authors:** Natalia Łukasik, Koleta Hemine, Iwona Anusiewicz, Piotr Skurski, Ewa Paluszkiewicz

**Affiliations:** 1 Department of Chemistry and Technology of Functional Materials, Faculty of Chemistry, Gdańsk University of Technology, 11/12 Narutowicza Street, 80-233 Gdańsk, Poland; 2 Laboratory of Quantum Chemistry, Faculty of Chemistry, University of Gdańsk, 63 Wita Stwosza Street, 80-308 Gdańsk, Poland; iwona.anusiewicz@ug.edu.pl (I.A.); piotr.skurski@ug.edu.pl (P.S.); 3 Department of Pharmaceutical Technology and Biochemistry, Faculty of Chemistry, Gdańsk University of Technology, 11/12 Narutowicza Street, 80-233 Gdańsk, Poland; ewa.paluszkiewicz@pg.edu.pl

**Keywords:** azobenzene, photoresponsive materials, photoisomerization, *cis-trans* thermal back-reaction

## Abstract

Azobenzene derivatives are one of the most important molecular switches for biological and material science applications. Although these systems represent a well-known group of compounds, there remains a need to identify the factors influencing their photochemical properties in order to design azobenzene-based technologies in a rational way. In this contribution, we describe the synthesis and characterization of two novel amides (L1 and L2) containing photoresponsive azobenzene units. The photochemical properties of the obtained compounds were investigated in DMSO by UV-Vis spectrophotometry, as well as ^1^H NMR spectroscopy, and the obtained results were rationalized via Density Functional Theory (DFT) methods. After irradiation with UV light, both amides underwent *trans* to *cis* isomerization, yielding 40% and 22% of the *cis* isomer of L1 and L2 amides, respectively. Quantum yields of this process were determined as 6.19% and 2.79% for L1 and L2, respectively. The reverse reaction (i.e., *cis* to *trans* isomerization) could be achieved after thermal or visible light activation. The analysis of the theoretically determined equilibrium structure of the transition-state connecting *cis* and *trans* isomers on the reaction path indicated that the *trans*-*cis* interconversion is pursued via the flipping of the substituent, rather than its rotation around the N=N bond. The kinetics of thermal back-reaction and the effect of the presence of the selected ions on the half-life of the *cis* form were also investigated and discussed. In the case of L1, the presence of fluoride ions sped the thermal relaxation up, whereas the half-life time of *cis*-L2 was extended in the presence of tested ions.

## 1. Introduction

The development of so-called smart materials, whose functions can be activated or inhibited on demand via external stimuli, is of growing interest for material science and biomedical applications [1]. The presence of a tunable unit within such a material enables controlling of the molecules shape and its resulting properties using, for instance, heat, redox potential, pH, or a magnetic/electric field [2,3,4]. In fact, light radiation appears to be the most appealing external stimulus, as it is easy to apply and control to assure the desired energy matching by choosing the appropriate wavelength. In contrast to chemicals, photons do not contaminate the studied material and have low or negligible toxicity. By adjusting its intensity, light can be regulated in a quantitative manner [5]. The ability to have an influence on the key properties of molecular systems with the use of light creates promising perspectives for energy conversion, medical applications, or the development of next-generation photofunctional materials [6]. Among the light-activated devices, materials based on diarylethenes [7,8], spiropyrans [9,10], and azobenzenes [1,5] are particularly interesting because of their ease of addressability and compatibility with light in a wide range of condensed phases and fast response times.

Azobenzene derivatives are widely used as dyes and pigments (60–70% of the world’s production of industrial dyes [11]) and pH indicators [12]. They are also commonly the building blocks of materials, having potential applications in areas of optical storage media [13], drug delivery [14], liquid crystals [15], nonlinear optics [16], and chemosensors [17]. Recently, the cytotoxicity and in vivo phototoxicity studies revealed that azobenzene derivatives are relatively safe to use in sunscreens and cosmetic applications [18]. The attractiveness of azo compounds results from their relatively simple synthesis, as well as a clean photoisomerization reaction involving a reversible, light-initiated switching of the azobenzene geometry between the planar *trans* (*E*) isomer and the bent metastable *cis* (*Z*) form [19,20]. Light-triggered isomerization changes not only the shape of azobenzene molecules, but also influence their chemical and physical properties, due to the different spatial arrangements of the aromatic moieties [21]. The thermodynamically stable *trans* azobenzene (~12 kcal mol^−1^) characterized by vanishing dipole moment isomerizes via irradiation with near-ultraviolet (UV) light (λ = 300–400 nm); the *cis* form is characterized by a dipole moment of ~3 D [22]. In addition, the isomerization reduces the separation between the carbon atoms at the *para* positions of the azobenzene rings from 9.0 Å in the *trans* form to 5.5 Å in the *cis* isomer.

Azobenzene type molecules show a high intensity π → π* band in the UV-region (~320 nm) related to the delocalized π-bonding and a low intensity n → π* band in the visible region (450 nm) formed due to the presence of lone electron pairs at nitrogen atoms [20,23,24]. Upon *trans* to *cis* isomerization, the π → π* absorption band decreases and shifts to shorter wavelengths, whereas the intensity of n → π* absorption band increases [25]. Reverse *cis*-*trans* isomerization may occur either thermally in the dark or by illumination with visible light (λ = 425–500 nm) [22,25,26]. In fact, the rate of this process strictly determines the potential applications of azo-bearing materials. Photochromophores with the long-life *cis* isomer and a slow *cis* to *trans* conversion are valuable units of materials when integrated into information storage [27], as well as tools for the investigation of particular processes in living cells [28] and the photocontrol of enzyme activity [29]. On the other hand, materials characterized by fast back-isomerization (*cis* to *trans*) are useful in the development of photochromic ion-channel blockers and other systems whose rapid conversion into the initial *trans* state does not require the use of additional light sources [30].

The kinetics of the isomerization process strongly depends on the chemical structure of the photoresponsive system: the electron density at particular levels, the position of energy levels of π_N=N_, π*_N=N_, and LP_N_ orbitals. In particular, the isomerization rate constant is related to the position and character (i.e., electron-withdrawing groups (EWG) or electron-donating groups (EDG)) of the substituents in the benzene rings. For instance, the presence of the amino or hydroxyl group at the *ortho* or *para* position causes the redshift of the π→π* transition band, which is the result of the LUMO (π*) energy level lowering. As a consequence, the half-life of the *cis* isomer is shorter than that of the unsubstituted azobenzene [25,31]. In the other case, azobenzenes bearing either alkyl or amide moieties are characterized by relatively long *cis* to *trans* conversion [22].

Taking the above into account, the understanding of the isomerization kinetics of substituted azo compounds is of great importance in order to gain control over photochemical properties and, thus, to design photosensitive materials for strictly accurate applications. For this reason, we decided to synthesize two photoresponsive amide derivatives of azobenzene-4,4′-dicarboxylic acid and perform their key experimental and theoretical studies. Since these compounds have not been described in the literature thus far, we believe the thorough investigation of the isomerization process involving the reported amides is essential for designing various azo-bearing photosensitive materials in the near future.

## 2. Materials and Methods

### 2.1. General

All chemicals of the highest available purity were purchased from commercial sources and used without further purification. The reaction progress was monitored by TLC using aluminum sheets covered with silica gel 60F_254_ (Merck). ^1^H NMR and ^13^C spectra were recorded on a Bruker Avance III HD (Karlsruhe, Germany) and a Varian Unity Inova 500 apparatus (Palo Alto, CA, USA) at 400, 500, and 100 MHz, respectively. Chemical shifts were reported as *δ* (ppm) values, in relation to TMS. FTIR spectra (KBr pellets) were taken on a Nicolet iS10 apparatus (Thermo Fisher Scientific, Waltham, MA, USA). Mass spectra were recorded using an Agilent 6470A triple quadrupole LC/MS system (Agilent Technology, Waldbronn, Germany) with electrospray ionization source (ESI) in a SCAN mode. Samples were prepared as 1 μg/mL solutions in DMSO and were supplied in 1 µL aliquots to the mass spectrometer in the mixture of acetonitrile (acetonitrile: water: formic acid (38:57:5 *v*/*v*/*v*)) at a flow rate of 500 μL/min. UV-Vis titrations were carried out in DMSO (POCH) using an UNICAM UV 300 spectrophotometer (Spectronic Unicam, Leeds, UK). For spectrophotometric measurements, 1 cm quartz cuvettes were used. UV irradiation experiments were carried out in a prototype photoreactor, designed by D. Wysiecki and constructed in cooperation with Enviklim Company (Gdańsk, Poland). The reactor is equipped with 3 UVA diode arrays (2 × UV-D6565-4LED, 40 W and 1 × UV-D6565-15LED, 150 W, λ = 365–370 nm). In the photoisomerization studies, a 100 W xenon solar light simulator with an AM 1.5 filter (LOT Quantum Design, Darmstadt, Germany) was used.

### 2.2. Synthesis and Characterization of the Compounds

#### 2.2.1. Compound L1

Azobenzene-4,4′-dicarboxylic acid dichloride (165 mg, 0.54 mmol) was gradually added to a round-bottom flask containing a magnetic stirred solution of *m*-aminophenol (117 mg, 1.07 mmol) in anhydrous acetone (10 mL) and triethylamine (0.15 mL, 1.07 mmol) used as a HCl scavenger. Subsequently, the reaction mixture was stirred under a reflux condenser for 12 h (temperature ~60 °C). The obtained precipitate was filtered off under reduced pressure and crystalized from acetone: propan-2-ol mixture (9:1, *v*/*v*).

**L1:** orange solid, 218 mg (90%), mp. 192–195 °C; TLC: R_f_ = 0.68 (dichloromethane: methanol, 15:2, *v*/*v*); ^1^H NMR (400 MHz, DMSO-*d_6_*, δ (ppm)): 6.53–6.56 (2H, dd, *J* = 7.9, 1.2 Hz), 7.13–7.22 (4H, m), 7.40 (2H, s), 8.06–8.08 (4H, m), 8.18–8.20 (4H, m), 9.47 (2H, s), and 10.34 (2H, s); ^13^C NMR (100 MHz, DMSO-*d_6_*, δ (ppm)): 108.0, 111.5, 111. 7, 123.1, 129.6, 129.7, 138.1, 140.5, 153.8, 158.0, and 165.1; IR (KBr pellet) cm^−1^: 3353, 3085, 1629, 1604, 1524, 1445, 1356, 1283, 1205, 866, 774, and 684; HRMS ESI(−), ([M − H]^−^): 451.0 for compound C_26_H_19_N_4_O_4_, calculated 451.5.

#### 2.2.2. Compound L2

Azobenzene-4,4′-dicarboxylic acid dichloride (150 mg, 0.49 mmol) was gradually added to a round-bottom flask containing a magnetic stirred solution of 2-amino-4-nitrophenol (151 mg, 0.98 mmol) in anhydrous dichloromethane (10 mL) and 4-dimethylaminopyridine (119 mg, 0.98 mmol) used as a HCl scavenger. Subsequently, the reaction mixture was stirred under a reflux condenser for 12 h (temperature ~40 °C). The obtained precipitate was filtered off under reduced pressure and crystalized from a fresh portion of dichloromethane.

**L2:** orange solid, 155 mg (58%), mp. 322–325 °C; TLC: R_f_ = 0.50 (dichloromethane: methanol, 15:2, *v*/*v*); ^1^H NMR (400 MHz, DMSO-*d_6_*, δ (ppm)): 6.99 (1H, d, *J* = 7.6 Hz), 7.14 (1H, d, *J* = 9.0 Hz), 8.01–8.25 (10H, m), 8.77 (1H, d, *J* = 2.8 Hz), 9.92 (1H, s), 11.66 (2H, s), and 13.30 (2H, s); ^13^C NMR (100 MHz, DMSO-*d_6_*, δ (ppm)): 107.4, 115.9, 119.9, 122.5, 123.3, 123.3, 126.4, 129.6, 131.2, 133.8, 137.0, 139.5, 139.6, 154.1, 154.6, 156.5, 165.2, and 167.1; IR (KBr pellet) cm^−1^: 3442, 3096, 1736, 1686, 1637, 1602, 1527, 1421, 1341, 1262, 1185, 870, 783, and 698; HRMS ESI(−), ([M − H]^−^): 541.1 for compound C_26_H_19_N_6_O_8_ calculated 541.4.

### 2.3. Theoretical Calculations

The equilibrium structures and energies of the L1 and L2 compounds in DMSO were determined by employing the Density Functional Theory (DFT) with the hybrid exchange–correlation functional CAM-B3LYP (the long-range-corrected version of B3LYP using the Coulomb-attenuating method [32]) together with the 6-311++G(d,p) [33,34] basis set. The effects of surrounding DMSO molecules were estimated by employing the polarized continuum solvation model (PCM) [35,36,37] within a self-consistent reaction field treatment, as implemented in the Gaussian 16 program (the default options for PCM and the dielectric constant of 46.826 for dimethyl sulfoxide were used). Electronic excitation energies for singlet states and the corresponding oscillator strengths were obtained (at the equilibrium geometry of the absorbing species) from the TD-DFT technique [38,39,40] using the same CAM-B3LYP functional and 6-311++G(d,p) basis set. The harmonic vibrational frequencies (unscaled) of their equilibrium structures were obtained at the same CAM-B3LYP/6-311++G(d,p) theory level to assure consistency. All of the calculations were performed with the Gaussian 16 program suite [41].

### 2.4. Photoisomerization Studies

UV irradiation experiments were carried out in a quartz cuvette (l = 1 cm) in DMSO. The progress of the photoisomerization was monitored by UV-Vis spectrophotometry (c ~ 10^−5^ M) and ^1^H NMR spectroscopy (c ~ 10^−3^ M). The reverse isomerization was led in darkness at 333.15 K or using a solar light simulator.

### 2.5. Ligand–Ion Interactions Studies

Complexation studies were performed via the UV-Vis titration of the ligands solution in DMSO with the respective metal perchlorates (Li^+^, Na^+^, K^+^, Mg^2+^, Ca^2+^, Sr^2+^, Ba^2+^, Co^2+^, Ni^2+^, Cu^2+^, Pb^2+^, and Zn^2+^) to study their interactions with metal cations and tetra-*n*-butylammonium (TBA) salts (halides, nitrate(V), hydrogen sulfate, thiocyanate, perchlorate, *p*-toluenesulfonate, benzoate BzO^−^, acetate AcO^−^, and dihydrogen phosphate) in order to test the interactions with anions. The stock solutions of ligands (~10^−4^ M) and metal perchlorates or TBA salts (~10^−2^ M) were prepared by weighing the respective quantities and dissolving them in DMSO in volumetric flasks. Titrations were carried out in a quartz cuvette with path length of 1 cm with a starting volume of the ligand solution equal to 2.3 mL. To suppress the deprotonation process, titrations were also carried out in the presence of acetic acid (20-fold molar excess, in relation to the ligand concentration). On the basis of the experimental data, the stability constant values were determined using BindFit v.05 software [42,43].

### 2.6. Complexes Preparation for Spectroscopic Experiments

Complexes for FTIR and ^1^H NMR experiments were prepared by dissolving the respective amide (0.004 mmol) and tetra-*n*-butylammonium fluoride (0.004 mmol) in a 15 mL of acetone: dichloromethane mixture (3:2, *v*/*v*). Subsequently, the mixture was stirred until complete dissolution. After solvent evaporation under reduced pressure, the FTIR spectra of the obtained samples were recorded. For the ^1^H NMR experiments, the samples were dissolved in 0.7 mL of DMSO-*d_6_* before analysis.

## 3. Results and Discussion

### 3.1. Synthesis and Characterization

**Scheme 1:**
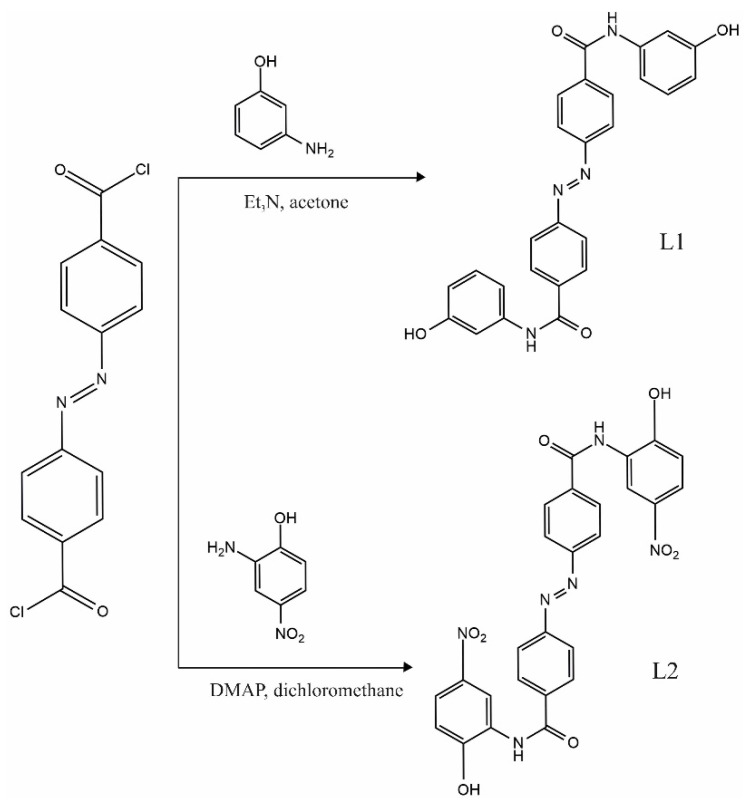
General synthetic route for L1 and L2 obtainment.

**Figure 1:**
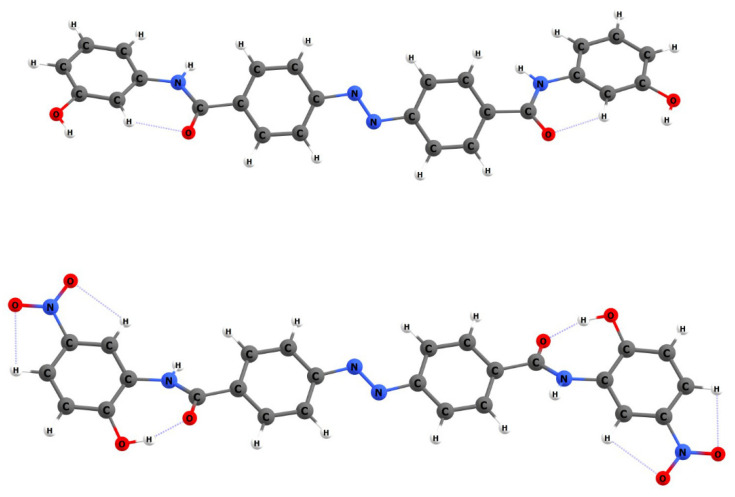
Theoretically predicted equilibrium structures of the lowest energy isomers of L1 (**top**) and L2 (**bottom**) compounds.

L1 and L2 compounds were obtained from azobenzene-4,4′-dicarboxylic acid dichloride, according to the procedure shown in Scheme 1. Detailed information concerning the spectroscopic characterization of the compounds can be found in the Materials and Methods section and the Supplementary Materials (SM: Figures S1 and S2). The chromogenic amides contain hydroxyl groups in peripheral rings, both of which, with amide (CO-NH) moieties, may serve as donors in the formation of hydrogen bonds. Additionally, the L2 compound contains electron-withdrawing nitro groups in its structure. Both considered azo compounds can exist in two isomeric forms: *trans* (*E*) and *cis* (*Z*). According to the calculations performed at the CAM-B3LYP/6-311++G(d,p) theory level, the most stable structures of L1 and L2 in DMSO solvent (whose effects were approximated by employing the polarized continuum solvation model, see Section 2.3 for details) correspond to the *trans* configurations (with respect to the N=N bond), see Figure 1 where these lowest energy isomers are depicted. In fact, we found six *trans* L1 conformers (L1_E_) and four *trans* L2 conformers (L2_E_) with relative energies within 6 kJ/mol (see Tables S1 and S2 and Figures S1 and S2 in the SM). Since these low energy L1_E_ and L2_E_ systems differ with one another only by the mutual orientation of their substituents, with respect to the central Ph-N=N-Ph moiety, one may expect their interconversion (via the rotation about the single C-C and C-N bonds), to likely be at room temperatures. As far as the *cis* isomers are concerned, the most stable *cis* L1 structure was predicted to be 53 kJ/mol higher in energy than the lowest energy *trans* L1, while the relative energy of the most stable *cis* L2 structure was calculated to be 52 kJ/mol (with respect to the lowest energy *trans* L2). In addition, we found other *cis* L1 (L1_Z_) and *cis* L2 (L2_Z_) conformers, whose formation from their corresponding, most stable *cis* structures might be considered likely, as their relative energies are larger by only 1–6 kJ/mol (Tables S1 and S2 and Figures S3 and S4).

In all studied *trans* and *cis* L1 and L2 systems, the N=N bond length was predicted to span the narrow 1.234–1.239 Å range and, thus, resembled a typical N=N double bond. The values of Wiberg Bond Indices (WBI) [44] calculated for the *trans* L1 (1.812), *cis* L1 (1.946–1.948), *trans* L2 (1.812–1.815), and *cis* L2 (1.946–1.951) seem to confirm that the N=N bond order approaches 2 in all considered L1 and L2 systems. In particular, the WBI values indicate that the double-bond character is more profound in the *cis* isomers, which is consistent with the slightly shorter (by 0.005 Å) N=N separations predicted for those species.

### 3.2. Isomerization Studies

**Figure 2:**
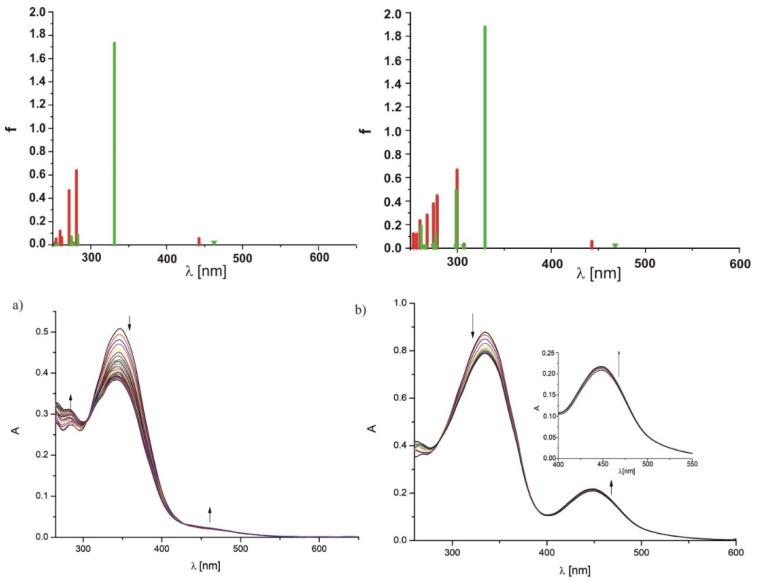
Changes in UV-Vis spectrum of (**a**) L1 (c = 3.24 × 10^−5^ M) and (**b**) L2 (c = 2.05 × 10^−5^ M) solution in DMSO upon irradiation with UV light (λ = 365 nm) (bottom panel) and the corresponding π,π* and n,π* transition energies predicted theoretically (green lines for trans isomer, red lines for cis isomer) (top panel).

The photochemical behavior of amides was studied by spectroscopic and theoretical methods. In the UV-Vis spectra of the compound solutions in DMSO, the changes upon UV light irradiation (λ = 365 nm) were observed (Figure 2). After 2 and 6 min of the UV irradiation of L1 and L2 solutions, respectively, a hypochromic effect in the region of the π→π* band and a less pronounced hyperchromic effect for n→π* band could be seen until a photostationary state was achieved. UV light-induced spectral changes were more profound for L1 and are characteristic for the *trans* to *cis* isomerization of azo compounds [45]. The determined quantum yield value of the photoconversion of L1 (see SM for details: Figure S5 [46,47]) is more than twice as large as for the nitro derivative L2 (Table 1), which indicates the higher efficiency of the *trans* to *cis* isomerization of L1.

In order to support the analysis of the UV-Vis spectra of L1 and L2 solutions in DMSO, we calculated (by employing time-dependent density functional theory method, TD-DFT, see Section 2.3 for details) the S_0_→S_i_ excitation energies with their corresponding oscillator strengths, see Tables S3 and S5. The comparison of theoretically predicted singlet-singlet π,π* and n,π* transition energies to the experimental absorption spectra in DMSO (Figure 2, Tables S3 and S5, and Figures S6 and S7) enabled the proper assignment of spectral features and their interpretation.

**Figure 3:**
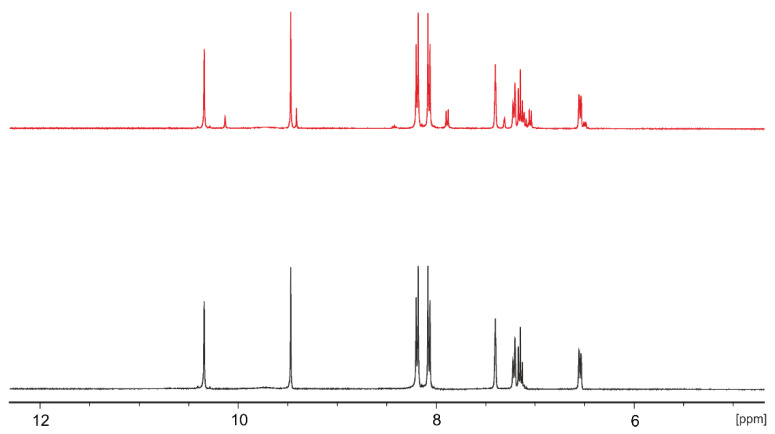
^1^H NMR spectra of L1 solution (17.7 mM) in DMSO-*d_6_* before (**bottom**) and after (**top**) UV-irradiation (λ = 365 nm).

On the basis of ^1^H NMR spectra, it was estimated that the irradiation of the compound solutions with UV light yielded 40% and 22% of a *cis* form of L1 and L2, respectively (see Figure 3, where the ^1^H NMR spectra of L1 are depicted as a representative example). The observed upfield shifts of OH, NH, and aromatic signals for the *cis*-enriched mixture of L1 and L2 indicated a characteristic effect of the magnetic shielding of the overlapping phenyl rings of the concaved *cis* form [48]. The most significant chemical shifts in the aromatic proton signal region could be seen for protons of azobenzene rings, which may be related to the light-induced change of the configuration.

**Figure 4:**
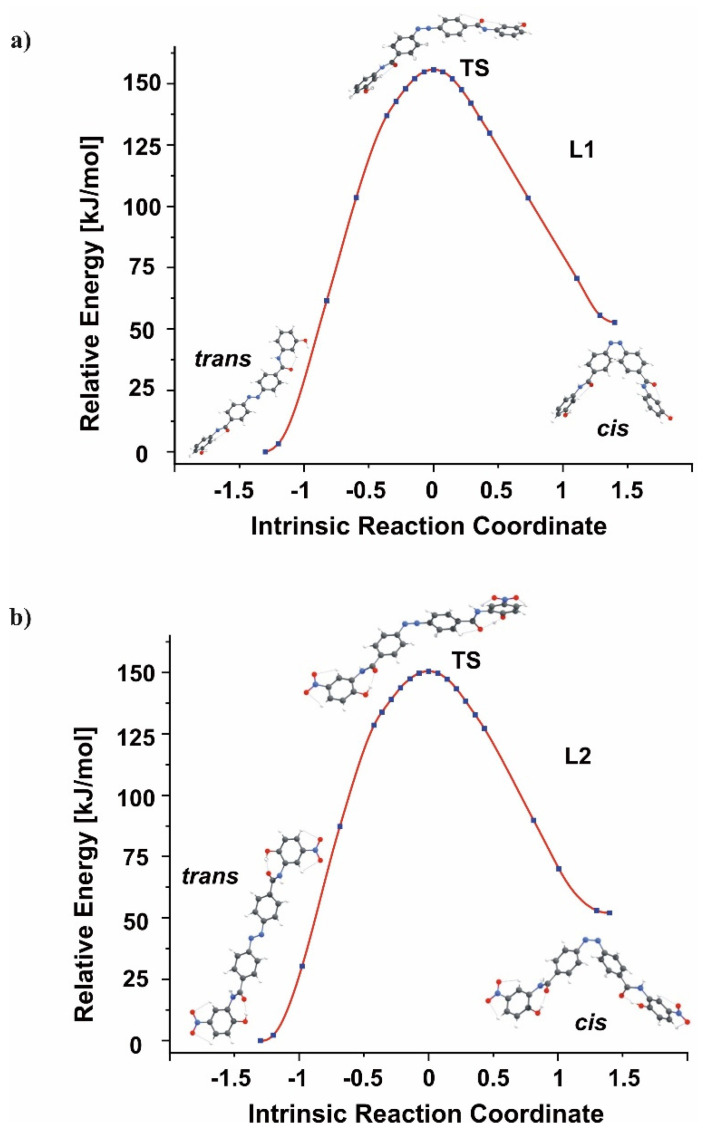
Theoretically predicted energy profiles (IRC paths) leading from the lowest energy *trans* L1 (**a**) and *trans* L2 (**b**) isomers over a transition state (TS) to the corresponding, most stable *cis* L1 and *cis* L2 isomer. The equilibrium structures of all stationary points involved in these processes are also shown.

The obtainment of the *cis*-enriched mixture upon UV-irradiation is consistent with the theoretically predicted energy profiles corresponding to the *trans*→*cis* isomerization of L1 and L2 depicted in Figure 4. Namely, *trans* and *cis* minimum energy structures are separated by the kinetic barriers whose heights (in the *trans*→*cis* direction) were evaluated to be 155.7 and 150.5 kJ/mol for L1 and L2, respectively. Clearly, surmounting such significant kinetic barriers would not be possible at room temperature without providing sufficient activation energy (as the rate constants of ca. 10^−15^–10^−14^ s^−1^ are predicted for these processes at T = 298.15 K).

A reverse process (i.e., *cis* to *trans* isomerization of L1) was possible upon the sample heating at 333.15 K in darkness, whereas the analogous back-isomerization of L2 nitro derivative was achieved after the sample irradiation with visible light. The *cis* to *trans* thermal transition of L1 followed the first-order kinetic model (Figure S8). The rate constant for the temperature induced *cis* to *trans* isomerization of L1 was determined to be 2.3 × 10^−6^ s^−1^; the half-life time of the *cis* isomer was estimated as 82.3 h at 298.15 K (98.4 min at 333.15 K), which indicates a larger stability of this form in comparison to the unsubstituted azobenzene (whose half-life time was estimated as equal to 2 days [26]). In order to determine the kinetic parameters of the reverse isomerization, the process was also studied at 323.15 K and 343.15 K (Figure S9). The values of activation energies, enthalpies, entropies, and Gibbs energies determined at 298.15 K are gathered in Table 2. The obtained results are consistent with data for bare and urea-substituted azobenzenes, described by Jurczak et al. [49].

After 4 min of the irradiation of the *cis*-enriched mixture of L2, with visible light, a hyperchromic effect in the region of π→π* band was observed, suggesting *cis* to *trans* isomerization. The reverse process (i.e., *trans* to *cis* isomerization), as it was mentioned before, required a longer time (6 min) to achieve the photostationary state.

**Figure 5:**
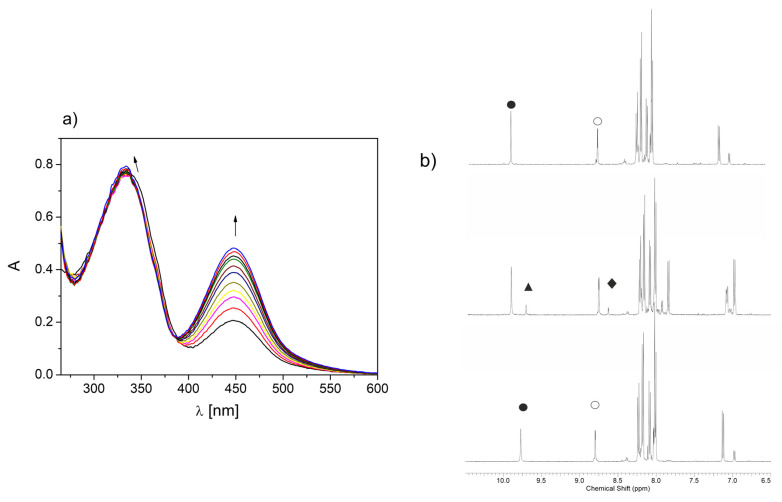
(**a**) Spectral changes registered upon heating at 333.15K of *cis*-enriched mixture of L2 in DMSO; (**b**) comparison of partial ^1^H NMR spectra of *trans*-L2 solution (c = 5.6 mM, top panel), *cis*-enriched mixture obtained upon UV irradiation (λ = 365 nm, middle panel), and solution heated after irradiation at 333.15 K (bottom panel) in DMSO-*d_6_*.

Heating the previously UV-irradiated solution of L2 in darkness caused the increase of the band at ca. 450 nm that corresponds to n→π* transition (Figure 5a). Slight hyperchromic effect in the region of π→π* band could also be seen; however, the final spectrum was not consistent with the spectrum of pure *trans*-L2. The ^1^H NMR spectra of the *trans*-L2, *cis*-enriched mixture obtained after irradiation with UV light and solution heated at 333.15 K (previously irradiated with UV light) are shown in Figure 5b. The signals characteristic for the *cis* isomer (such as ▲ and ♦ data points at 9.7 and 8.6 ppm, respectively, in the spectrum shown in the middle panel of Figure 5b) were absent in the spectrum recorded after the sample heating at 333.15 K (the bottom panel of Figure 5b), suggesting that thermal back-isomerization occurred. The shape and intensity of aromatic protons signals in the spectrum registered at higher temperature correspond to the spectrum of *trans*-L2 (presented in the top panel of Figure 5b), however, some differences could be noted. Namely, the signal of a proton located in the *ortho* position, with respect to nitro and amide moieties from one peripheral ring (●) of L2, was shifted to lower ppm values (Δδ = −0.17 ppm) at higher temperature, whereas the proton signal related to the second ring (○) was slightly shifted downfield (Δδ = 0.03 ppm), in comparison to the spectrum registered at 298.15 K. The observed shielding effect is likely a consequence of intramolecular CO∙∙∙OH hydrogen bonds breaking, induced by thermal energy provided to the system. Such an interpretation is also supported by the analysis of the UV-Vis spectrum of L2 solution, in which the significant hyperchromic effect observed during the sample heating is associated with heteroatoms, such as oxygen and nitrogen (the region of n→π* transition band).

According to the results obtained from spectrophotometric measurements, it was concluded that the thermal isomerization accompanying intramolecular hydrogen bonds breaking is a first-order reaction, with the rate constant k equal to 3.6 × 10^−5^ s^−1^ and the half-life time of the *cis* isomer estimated as 5.4 h at 298.15 K (15 min at 333.15 K) (Figure S10). The kinetic parameters of the temperature-induced change of L2 configuration are summarized in Table 2. In comparison to L1, thermal back-isomerization of the L2 solution requires lower activation energy. More negative value of the activation entropy determined for nitro derivative transition may result from the compilation of two processes, namely, *cis* to *trans* isomerization, and the breaking of intramolecular hydrogen bonds. The lower stability of *cis*-L2 than *cis*-L1 may be connected with the presence of nitro substituents in the benzene rings of L2. The electron-withdrawing effect of these moieties leads to a push-pull system, which is described to cause unstable *cis* isomers of azobenzene derivatives [50,51].

Theoretical calculations showed that *cis→trans* isomerization required overcoming a much smaller, yet substantial, kinetic barrier of 103.0 and 98.4 kJ/mol for L1 and L2, respectively. Since the corresponding Gibbs activation energies (ΔG^‡^) at T = 298.15 K predicted theoretically (98.4 kJ/mol and 93.8 kJ/mol for L1 and L2, respectively) agree relatively well with those obtained experimentally (see Table 2), the rate constants (3.6 × 10^−5^ s^−1^ for L1 and 2.0 × 10^−4^ s^−1^ for L2) calculated for these reverse isomerization processes using the above mentioned ΔG^‡^ values seem reliable. In addition, we would like to point out that in both L1 and L2, cases the equilibrium structure of the transition state (containing a nearly linear N=N–X fragment, where X stands for a substituent) and the analysis of the imaginary vibration (primarily involving the bending of the N=N–X valence angle) indicates that the *trans*↔*cis* interconversion is pursued by the flipping of the X substituent, rather than its rotation about the N=N bond, see the transition state structures (labeled TS) depicted in Figure 4.

### 3.3. Interactions with Ions

**Figure 6:**
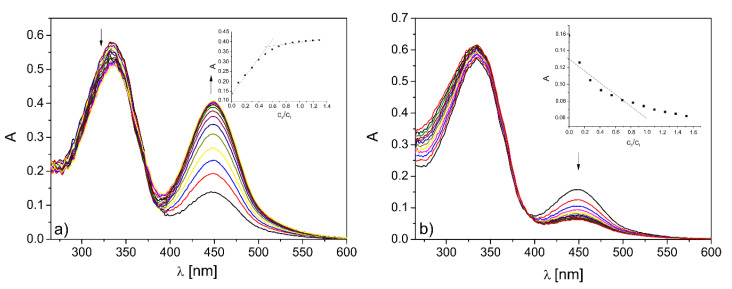
UV-Vis spectra of L2 solution (c = 1.62 × 10^−5^ M) in DMSO registered in the presence of (**a**) tetra-*n*-butylammonium benzoate (c = 0–2.57 × 10^−5^ M) and (**b**) copper(II) perchlorate (c = 0–3.02 × 10^−5^ M); insert: molar ratio plots.

Due to the presence of NH and OH groups serving as the moieties enabling the formation of hydrogen bonds, L1 and L2 compounds may interact with ions. In fact, such potential host-guest interactions may have an influence on the thermal relaxation of azo compounds [49,52]. Among the ions tested (see Materials and Methods section for details), the changes in UV-Vis spectrum of the L1 solution in DMSO were observed only in the presence of fluoride anions (Figure S10a). In the case of L2 compound, however, the changes were registered in the presence of fluorides, acetates, dihydrogen phosphates (Figure S10b–d), benzoates (as TBA salts), as well as copper(II) cations (in the form of perchlorate salt). The examples of spectrophotometric titrations are shown in Figure 6. In the presence of anions, a significant hyperchromic effect at ca. 450 nm (n→π* transition band) in L2 solution spectrum was observed. On the other hand, copper(II) cations caused an opposite effect, namely, a decrease of the band intensity was recorded. On the basis of the data obtained from spectrophotometric titrations, the stoichiometries and binding constant values of possible complexes were determined and presented in Table 3.

**Figure 7:**
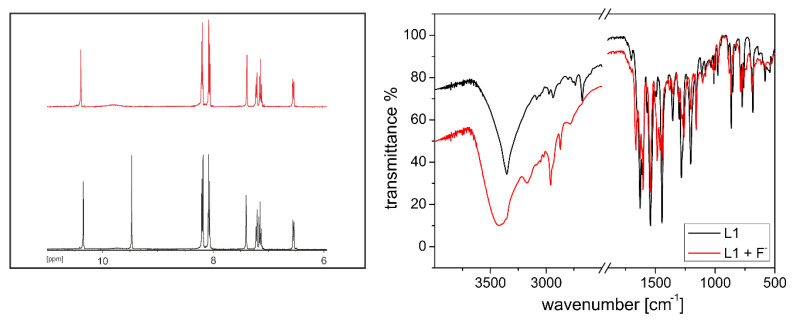
The comparison of ^1^H NMR (400 MHz, DMSO-*d_6_*, **left**) and FTIR (KBr pellet, **right**) spectra of L1 in the presence of equimolar amount of tetra-*n*-butylammonium fluoride.

Among the anions tested, the species exhibiting 1:1 stoichiometry are formed by L2 and fluoride, acetate, or copper(II) ions, whereas 1:2 (S:A) stoichiometry is more probable when L2 interacts with benzoate or dihydrogen phosphate anions. Among the last mentioned, the nitro derivative interacts more strongly with the Y-shaped carboxylic anions. As strongly basic anions in DMSO may cause the deprotonation of amide based ligands, the interactions of L1 and L2 compounds with ions were tested in the presence of the excess of acetic acid, which was used to suppress potential deprotonation [53,54]. Spectral changes observed after the addition of strongly basic fluoride anions were even noticed in the presence of the acid, which suggests that complex formation in the solution is more favorable than the deprotonation of the compounds. The evidence of L1 interactions with fluoride can also be found in ^1^H NMR and FTIR spectra (Figure 7). Namely, the OH signal seen in the ^1^H NMR of free amide L1 at 9.47 ppm was broadened and shifted downfield (Δδ ~ 0.33 ppm) in the presence of fluoride anions, thus suggesting the involvement of hydroxyl moieties in the fluoride ion binding (via the formation of H-bonds). The signal of NH protons was slightly shifted to higher ppm values (Δδ = 0.04 ppm), which can be connected with host-guest interactions. In the FTIR spectra of L1 recorded in the presence of fluoride ions, the band at ca. 3400 cm^−1^ ascribed to the stretching vibrations of the OH and NH groups was broadened and shifted to higher wavenumbers (by 68 cm^−1^) in comparison to the spectrum of free amide. The amide I band (seen in the L1 spectrum at 1629 cm^−1^) was split in the complex spectrum and shifted to higher wavenumbers. The shape and intensity of the bands corresponding to the C-O stretching vibrations at 1280–1200 cm^−1^ were changed in the presence of the anion. The observed changes may be a consequence of the heteroatoms involvement in the interactions with fluoride ions.

**Figure 8:**
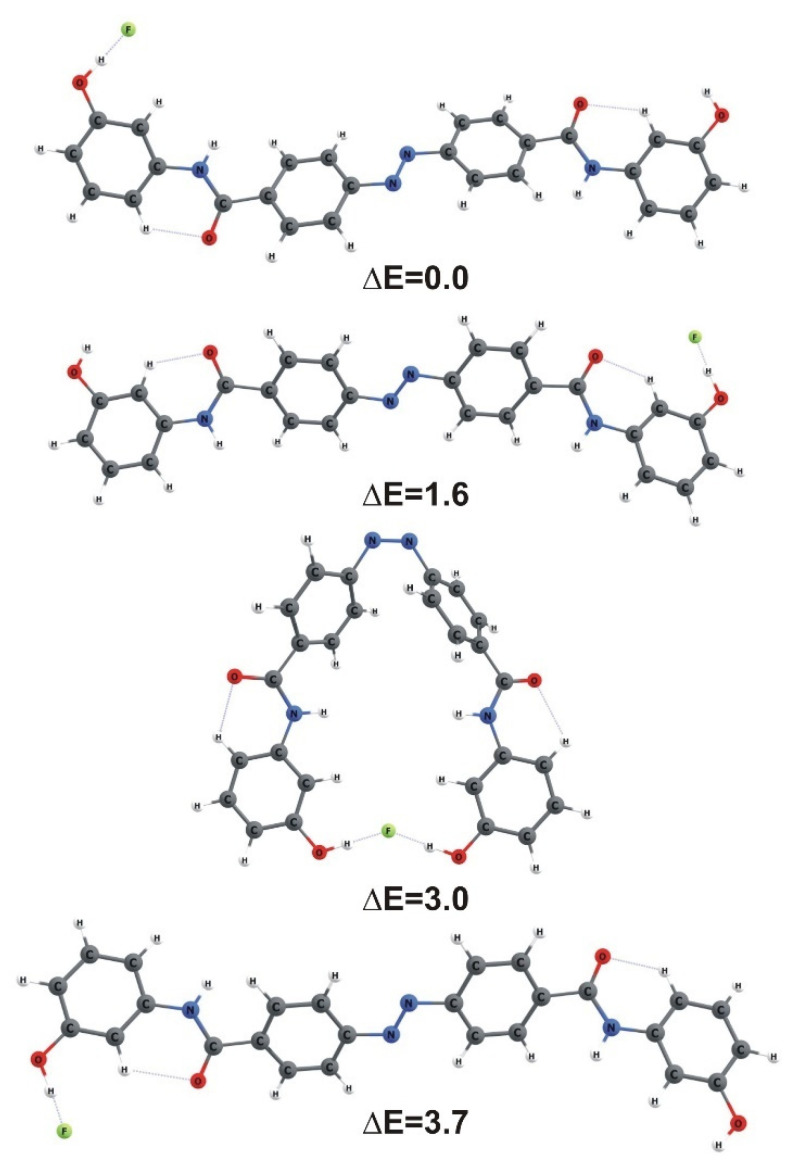
Theoretically predicted equilibrium structures of the lowest energy isomers formed upon an F^-^ attachment to L1. Relative energies (ΔE) are given in kJ/mol.

In the ^1^HMR spectrum of the UV-irradiated solution of L1, the shifts of signals ascribed to the protons of the *cis* form in the presence of fluoride anions were seen (Figure S11). The characteristics of the changes were similar to those observed for the pure *trans* isomer (i.e., downfield shift of the NH and OH protons signals), suggesting the involvement of the *cis* form in the interactions with the anion. On the basis of spectrophotometric titration, it was determined that under measurement conditions, the species of 1:2 stoichiometry (S:A) were formed (Figure S12a). This reveals that the application of UV irradiation influenced the binding mode (see Table 3 for comparison). Theoretical calculations revealed that the F^−^ anion attaches to the OH functional group via the OH∙∙∙F^-^ hydrogen bond, see Figure 8. Since the terminal hydroxyl groups in the *cis* L1 isomer are located closer to each other than in the *trans* L1 isomer, the bifurcated OH∙∙∙F^−^∙∙∙HO hydrogen bond can be formed in the former case. As a consequence, the *cis* L1 is more strongly stabilized by the attachment of F^-^ than the *trans* L1 which manifests itself by a small relative energy of the former (3 kJ/mol), with respect to the latter. Recalling that the relative energy of *cis* L1 (in the absence of F^−^), with respect to *trans* L1, was predicted to be substantially larger (53 kJ/mol) than the relative energy of *cis* L1 (in the presence of F^−^), with respect to *trans* L1/F^−^ (3 kJ/mol); one may conclude that the attachment of F^−^ renders the *cis* and *trans* isomers of L1 nearly isoenergetic.

For the L2 solution titration with TBAF, no significant differences between the stoichiometry before and after irradiation of the solution with the UV light were determined (Figure S12b). As revealed by our calculations, the presence of fluoride leads to a spontaneous proton withdrawal from the L2 compound (the resulting L2^−^∙∙∙HF complex consists of the negatively charged L2 and HF molecules connected via the hydrogen bond). This finding seems to be supported by the analysis of the FTIR spectrum registered for L2 in the presence of fluoride ions (Figure S13). The bands corresponding to stretching vibrations of carbonyl and C-O groups seen in the spectrum of free L2 at 1736 cm^−1^ and in the range of 1000–1200 cm^−1^, in the spectrum registered in the presence of fluoride anions are missing, which may suggest deprotonation of OH moieties. Although interactions in the solid state may differ from these in solution, the value of the F^−^-L2 stability constant reported in the Table 3 may describe deprotonation process.

The introduction of energy, in the form of heat, to the system increased the affinity of L2 towards ions (Table 4). It is possible that after the breaking of the hydrogen bonds between the NH and OH groups, these moieties became more prone to interaction with ions. The significant increase of stability constant values was determined for L2-BzO^−^ and L2-H_2_PO_4_^−^ systems at 333.15 K, in comparison with the values obtained from the titrations at room temperatures (in both cases ΔlogK ~ 2). The stoichiometry of the formed species with fluoride and copper(II) ions has changed at increased temperature, which suggests a different mode of amide-ion interactions (Figure S14).

The presence of tested ions influenced the rate of the thermal *cis* to *trans* isomerization of L1 and L2 solutions (Table 5). After the addition of an equimolar amount of fluoride ions to the L1 solution, the *cis* to *trans* isomerization sped up, likely as a consequence of the increased repulsion on the azo group, due to electron density transfer from the anion to the amide. In the case of the L2 thermal back-reaction, an opposite effect was observed. Ions interacting with the nitro derivative at the increased temperature caused a decrease in the reverse isomerization rate. As it was mentioned before, at increased temperature the intramolecular hydrogen bonds in L2 are broken and the amide has a higher affinity to tested ions. It is possible that under the measurement conditions, the (at 333.15 K) ions stabilized the *cis* isomer obtained after thermal activation.

## 4. Conclusions

The new amide derivatives of azobenzene-1,4-dicarboxylic acid were obtained in good (L1) and moderate (L2) yields using a facile synthetic protocol. On the basis of the ^1^H NMR spectra and theoretical calculations, it was concluded that in DMSO, the most stable form of L1 is a symmetric *trans* isomer, whereas for nitro derivative L2, the most probable structure corresponds to a *trans* form, with the peripheral rings rotated by 180 degrees, with respect to each other. The UV-irradiation of both azo-bearing compounds induced *trans* to *cis* isomerization. The photoisomerization process of L1 resulted in a higher conversion to the corresponding *cis* isomer than in the case of L2 (40% vs. 22%). The quantum yield of the *trans* to *cis* isomerization of L2 was over two times lower than for L1. The comparison of the half-life times of the respective *cis*-amides showed the higher stability of *cis*-L1 than *cis*-L2 and unsubstituted azobenzene. The return to a pure *trans* form of L1 was possible after heating the sample in darkness, whereas, in the case of L2, this thermal activation also caused the breaking of intramolecular hydrogen bonds. The rate of thermal back-isomerization could be altered by the presence of ions. Fluoride ions interacting with L1 via hydrogen bridges caused faster *cis* to *trans* relaxation. In the case of L2, the presence of tested ions decreased the *cis* to *trans* isomerization rate, probably due to the increased affinity of the L2 form obtained after thermal activation towards ions. The results of the theoretical calculations performed for the L1 and L2 species revealed not only the equilibrium structures of their most stable isomers, but also those corresponding to the low energy isomeric forms (together with their relative energies). The analysis of the intrinsic reaction path connecting the *trans* and *cis* isomers of both the L1 and L2 systems resulted in the recognizing that the *trans-cis* interconversion process is pursued by the flipping of a substituent, rather than its rotation about the N=N bond.

## Figures and Tables

**Table 1:** Spectroscopic data obtained upon irradiation of L1 and L2 solution in DMSO with UV light (λ = 365 nm); ΔA—absorbance difference at the π→π* band.

Compound	λ_max *E*_ (nm)		ε*_E_*(M^−1^ cm^−1^)	ΔA (%)	Φ*_E_*_-*Z*_ (%)
	π→π*	n→π*			
L1	347	~460	1.3 × 10^4^	25.0	6.19 ± 0.13
L2	335	448	3.1 × 10^4^	10.1	2.79 ± 0.22

**Table 2:** The activation energies (E_a_), activation enthalpies (ΔH ^‡^), activation entropies (ΔS ^‡^), and activation Gibbs energies (ΔG ^‡^) for thermal *cis* to *trans* isomerization of L1 and L2 in DMSO. All values are given in kJ/mol, beside ΔS ^‡^, which is given in J/molK.

Compound	E_a_	ΔH ^‡^	ΔS ^‡^	ΔG ^‡^
L1	98.6 ± 1.8	95.8 ± 1.4	−31.2 ± 2.8	105.1 ± 1.4
L2	70.9 ± 0.7	68.2 ± 1.1	−101.2 ± 3.4	98.4 ± 4.4

**Table 3:** The stoichiometry (salt:amide) and values of stability constants (logK) for amides L1 and L2 with ions (as TBA or perchlorate salts) in DMSO.

Stoichiometry (S:A)	L1	L2				
	F^−^	F^−^	AcO^−^	BzO^−^	H_2_PO_4_^−^	Cu^2+^
1:1		5.65 ± 0.03	5.63 ± 0.12			5.66 ± 0.07
1:2				4.51 ± 0.01	4.38 ± 0.09	
2:1	5.22 ± 0.57					

**Table 4:** The stoichiometry (salt: amide) and values of stability constants (logK) for amide L2 with ions (as TBA or perchlorate salts) in DMSO at 333.15 K.

Stoichiometry (S:A)	F^−^	AcO^−^	BzO^−^	H_2_PO_4_^−^	Cu^2+^
1:1		5.76 ± 0.07			
1:2	9.15 ± 0.05		6.31 ± 0.04	6.21 ± 0.11	9.87 ± 0.16

**Table 5:** The half-life time values (in minutes) of *cis* to *trans* thermal relaxation, at 333.15 K in darkness, of L1 and L2 solutions in DMSO in the presence of equimolar amount of ions.

Sample Code	t_1/2_ (Free)	t_1/2_ (F^−^)	t_1/2_ (AcO^−^)	t_1/2_ (BzO^−^)	t_1/2_ (H_2_PO_4_^−^)	t_1/2_ (Cu^2+^)
L1	94	41	-	51	-	-
L2	15	49	47	62	42	52

## Data Availability

Data Sharing is not applicable.

## Supplementary Materials

The following are available online at https://www.mdpi.com/article/10.3390/ma14143995/s1, Figure S1: ^1^H (top) and ^13^C NMR (bottom) spectra of L1 in DMSO-*d_6_*; Figure S2: ^1^H (top) and ^13^C NMR (bottom) spectra of L2 in DMSO-*d_6_*; Figure S3: The CAM-B3LYP/6-311++G(d,p) equilibrium structures of L1_E_ and L1_Z_ isomers; Figure S4: The CAM-B3LYP/6-311++G(d,p) equilibrium structures of L2_E_ and L2_Z_ isomers; Figure S5: Concentration of (**a**) Fe^2+^ ions, (**b**) *cis*-L1, (c = 7.33 × 10^−5^ M), and (**c**) *cis*-L2 (c = 7.62 × 10^−5^ M) as a function of time (s) during irradiation with UV light (λ = 365 nm); Figure S6: Highest occupied molecular orbitals (HOMO) and lowest unoccupied molecular orbitals (LUMO) for the ground singlet state of L1_E_(a) and L1_Z_(a). The LP orbital for L1_E_(a) is also presented; Figure S7: Highest occupied molecular orbitals (HOMO) and lowest unoccupied molecular orbitals (LUMO) for the ground singlet state of L2_E_(a) and L2_Z_(a). The LP orbital for L2_E_(a) is also presented; Figure S8: The first-order reaction plot obtained for *cis*-enriched L1 solution in DMSO (3.24 × 10^−5^ M) at (**a**) 323 K, (**b**) 333 K, and (**c**) 343 K in darkness; Figure S9: The first-order reaction plot obtained for *cis*-enriched L2 solution in DMSO (2.05 × 10^−5^ M) at (**a**) 333.15 K, (**b**) 323.15 K, (**c**) 343.15 K in darkness, and (**d**) the Arrhenius plot obtained for L2 solution in DMSO; Figure S10: Spectral changes registered upon (**a**) L1 solution (c = 3.23 × 10^−5^ M) titration with tetra-*n*-butylammonium fluoride (c = 0–6.57 × 10^−4^ M), (**b**) L2 solution (c = 2.04 × 10^−5^ M) titration with tetra-*n*-butylammonium fluoride (c = 0–4.36 × 10^−5^ M), (**c**) L2 solution (c = 1.62 × 10^−5^ M) titration with tetra-*n*-butylammonium acetate (c = 0–3.11 × 10^−5^ M), and (**d**) L2 solution (c = 1.62 × 10^−5^ M) titration with tetra-*n*-butylammonium dihydrogen phosphate (c = 0–1.87 × 10^−5^ M) in DMSO; Figure S11: ^1^H NMR spectra of *cis*-enriched mixture of L1 (c = 17.7 mM, bottom, black) and in the presence of equimolar amount of tetra-*n*-butylammonium fluoride (top, red) registered in DMSO-*d_6_*; Figure S12: Spectral changes registered upon titration of *cis*-enriched mixture of (**a**) L1 solution (c = 6.18 × 10^−5^ M) with tetra-*n*-butylammonium fluoride (c = 0–1.04 × 10^−4^ M) and (**b**) L2 solution (c = 1.62 × 10^−5^ M) with tetra-*n*-butylammonium fluoride (c = 0–4.64 × 10^−4^ M) after UV-light irradiation (λ = 365 nm); Figure S13: Comparison of FTIR spectra (KBr pellet) of L2 and its mixture with equimolar amount of tetra-*n*-butylammonium fluoride; Figure S14: Spectral changes registered upon titration of L2 solution (**a**) (c = 2.41 × 10^−5^ M) with tetra-*n*-butylammonium fluoride (c = 0–3.33 × 10^−5^ M), (**b**) (c = 2.41 × 10^−5^ M) with tetra-*n*-butylammonium acetate (c = 0–3.18 × 10^−5^ M), (**c**) (c = 1.62 × 10^−5^ M) with tetra-*n*-butylammonium dihydrogen phosphate (c = 0–1.87 × 10^−5^ M), and (**d**) (c = 1.62 × 10^−5^ M) with tetra-*n*-butylammonium benzoate (c = 0–1.79 × 10^−5^ M), (**e**) (c = 1.70 × 10^−5^ M) with copper(II) perchlorate (c = 0–2.49 × 10^−5^ M) in DMSO at 333.15 K; Table S1: Relative electronic energies (ΔE in kJ/mol) with zero-point corrections (ΔE + ZPE in kJ/mol] and Gibbs free energies at T = 298.15 K (ΔG in kJ/mol) of the systems studied, with respect to their most stable isomers. The selected interatomic distances (in Å) and dihedral angles (in degrees) together with dipole moments (in Debye) and Wiberg Bond Indexes (WBI), calculated for the N=N bond, are also presented. The results are obtained at the CAM-B3LYP/6-311++G(d,p) level in DMSO; Table S2: Relative electronic energies (ΔE in kJ/mol) with zero-point corrections (ΔE+ZPE in kJ/mol] and Gibbs free energies at T = 298.15 K (ΔG in kJ/mol) of the systems, studied with respect to their most stable isomers. The selected interatomic distances (in Å) and dihedral angles (in degrees) together with dipole moments (in Debye) and Wiberg Bond Indexes (WBI), calculated for the N=N bond, are also presented. The results are obtained at the CAM-B3LYP/6-311++G(d,p) level in DMSO; Table S3: Predicted (TD-DFT CAM-B3LYP/6-311++G(d,p) in DMSO) singlet (*S*_0_→*S**_i_*) excitation energies, starting from the ground state of L1_E_(a) and L1_Z_(a) isomers, with their corresponding oscillator strengths, f; Table S4: Cartesian coordinates (in Å) of the ground state equilibrium structures of L1_E_(a) and L1_Z_(a); Table S5: Predicted (TD-DFT CAM-B3LYP/6-311++G(d,p) in DMSO) singlet (*S_0_*→*S_i_*) excitation energies starting from the ground state of L2_E_(a) and L2_Z_(a) isomers, with their corresponding oscillator strengths, f; Table S6: Cartesian coordinates (in Å) of the ground state equilibrium structures of L2_E_(a) and L2_Z_(a).
