# Supplementary material for: Photoresponsive Amide-Based Derivatives of Azobenzene-4,4′-Dicarboxylic Acid—Experimental and Theoretical Studies
Source: Materials (Basel). 2021 Jul 16;14(14):3995. doi: 10.3390/ma14143995 (PMC8306546; doi:10.3390/ma14143995)
Supplement: Supplementary file 1 [file materials-14-03995-s001.zip › materials-1275605-supplementary.pdf]

materials

MDPI

Supplementary Materials

Photoresponsive Amide-Based Derivatives of Azobenzene-4,4′-Dicarboxylic Acid—Experimental and Theoretical Studies

Natalia Łukasik [1,*], Koleta Hemine [1,*], Iwona Anusiewicz [2], Piotr Skurski [2] and Ewa Paluszkiewicz [3]

[1] Department of Chemistry and Technology of Functional Materials, Faculty of Chemistry, Gdańsk University of Technology, 11/12 Narutowicza Street, 80-233 Gdańsk, Poland
[2] Laboratory of Quantum Chemistry, Faculty of Chemistry, University of Gdańsk, 63 Wita Stwosza Street, 80-308 Gdańsk, Poland; iwona.anusiewicz@ug.edu.pl (I.A.); piotr.skurski@ug.edu.pl (P.S.)
[3] Department of Pharmaceutical Technology and Biochemistry, Faculty of Chemistry, Gdańsk University of Technology, 11/12 Narutowicza Street, 80-233 Gdańsk, Poland; ewa.paluszkiewicz@pg.edu.pl
* Correspondence: natalia.lukasik@pg.edu.pl (N.Ł.); koleta.hemine@pg.edu.pl (K.H.); Tel.: +58-347-2569 (N.Ł. & K.H.)

Citation: Łukasik, N.; Hemine, K.; Anusiewicz, I.; Skurski, P.; Paluszkiewicz, E. Photoresponsive Amide-Based Derivatives of Azobenzene-4,4′-Dicarboxylic Acid—Experimental and Theoretical Studies. *Materials* **2021**, *14*, 3995. https://doi.org/10.3390/ma14143995

Academic Editor: Dong Xiang

Received: 10 June 2021
Accepted: 13 July 2021
Published: 16 July 2021

Publisher's Note: MDPI stays neutral with regard to jurisdictional claims in published maps and institutional affiliations.

Copyright: © 2021 by the authors. Licensee MDPI, Basel, Switzerland. This article is an open access article distributed under the terms and conditions of the Creative Commons Attribution (CC BY) license (http://creativecommons.org/licenses/by/4.0/).

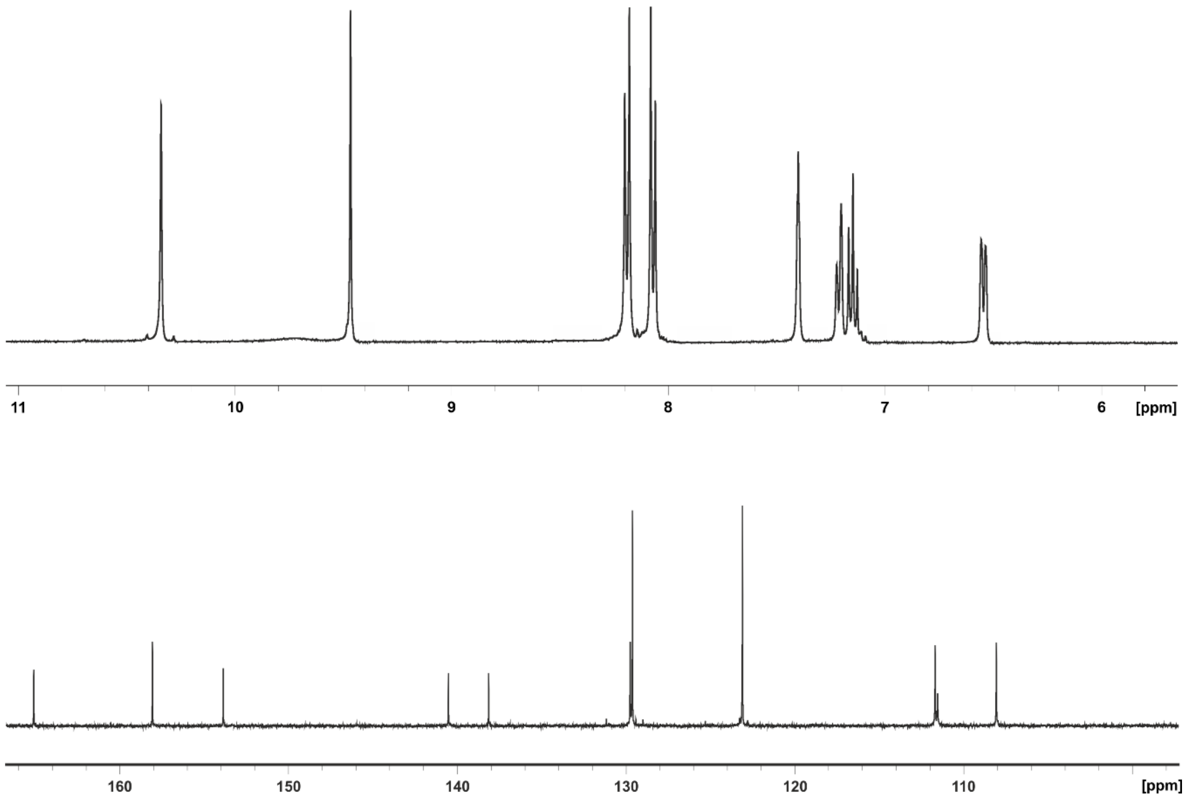

Figure S1. ^{1}H (top) and ^{13}C NMR (bottom) spectra of L1 in DMSO-d_6.

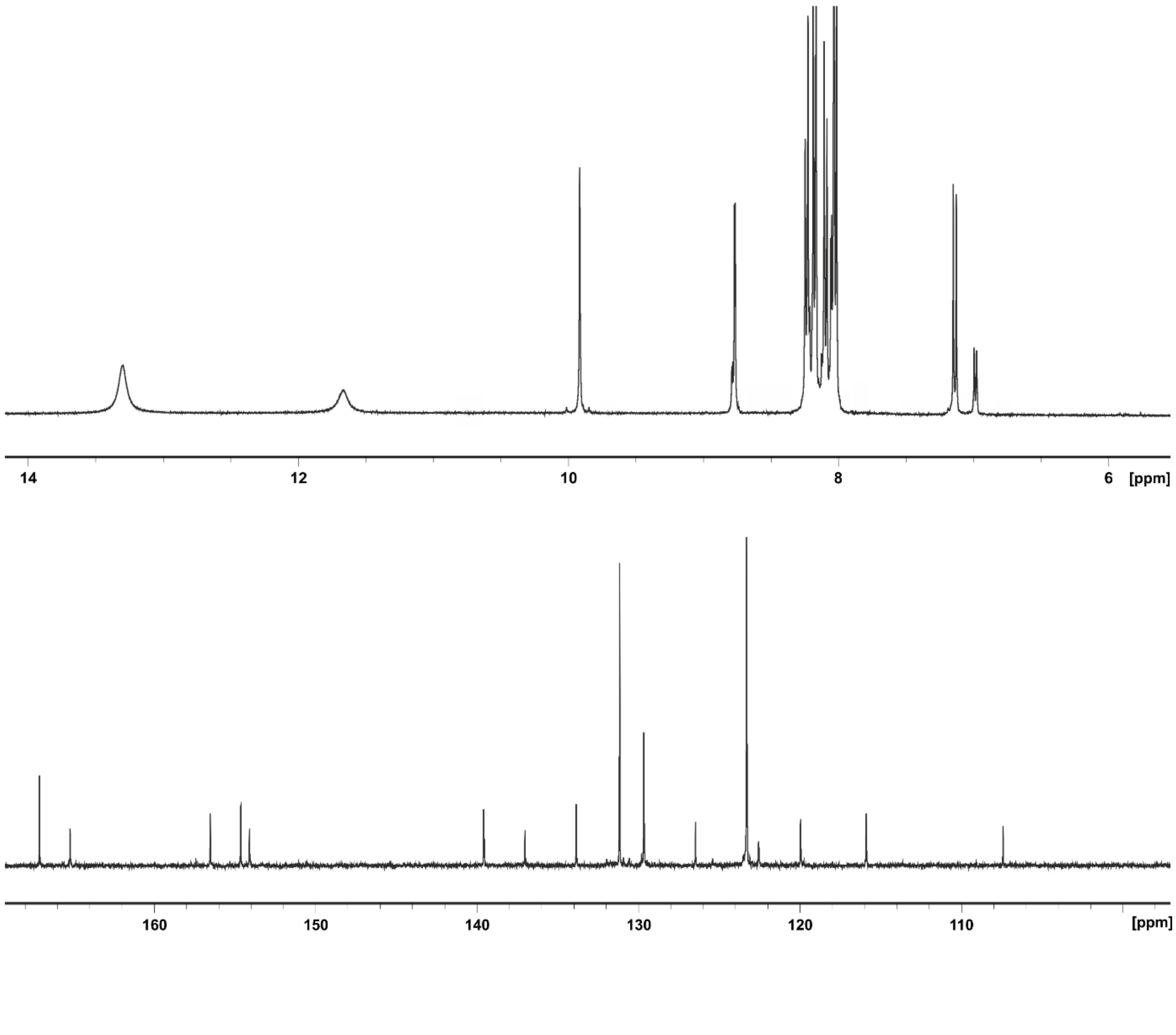

Figure S2. ^{1}H (top) and ^{13}C NMR (bottom) spectra of L2 in DMSO-*d*6.

Table S1. Relative electronic energies (ΔE in kJ/mol), relative electronic energies with zero-point corrections (ΔE+ZPE in kJ/mol] and Gibbs free energies at T=298.15K (ΔG in kJ/mol) of the systems studied with respect to their most stable isomers. The selected interatomic distances (in Å) and dihedral angles (in degrees) together with dipole moments (in Debye) and Wiberg Bond Indexes (WBI) calculated for the N=N bond are also presented. The results are obtained at the CAM-B3LYP/6-311++G(d,p) level in DMSO.

Species	ΔE	ΔE+ZPE	ΔG	r(CO…CO)	r(N=N)/WBI	∢CNNC	μ
**$L1_E$(a)**	**0**	0	0	12.038	1.239/1.812	179.8	4.09
$L1_E$(b)	0.11	-0.11	6.23	12.049	1.239/1.812	179.6	0
$L1_E$(c)	2.12	1.8	8.43	12.038	1.239/1.812	180	5.04
$L1_E$(d)	2.15	2.69	2.11	12.05	1.239/1.812	179.9	0.97
$L1_E$(e)	4.12	4.75	4.91	12.039	1.239/1.812	180	6
$L1_E$(f)	4.2	4.85	4.68	12.049	1.239/1.812	180	0
**$L1_Z$(a)**	52.7	52.74	56.66	8.134	1.235/1.946	7.2	4.28
$L1_Z$(b)	52.8	52.46	54.59	8.172	1.234/1.948	7.2	1.79
$L1_Z$(c)	54.8	55.28	59.31	8.165	1.235/1.947	7.1	2.33
$L1_Z$(d)	54.86	55.13	59.04	8.15	1.234/1.947	7.4	5.25
$L1_Z$(e)	56.78	57.26	60.51	8.19	1.234/1.947	7.1	2.79
$L1_Z$(f)	57.03	57.05	59.62	8.162	1.235/1.947	7.1	6.21

$L1_E$(a)

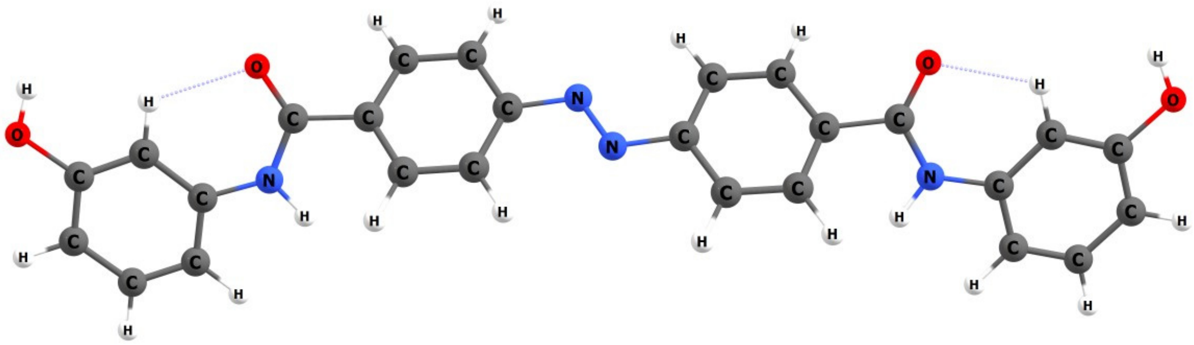

$L1_E$(b)

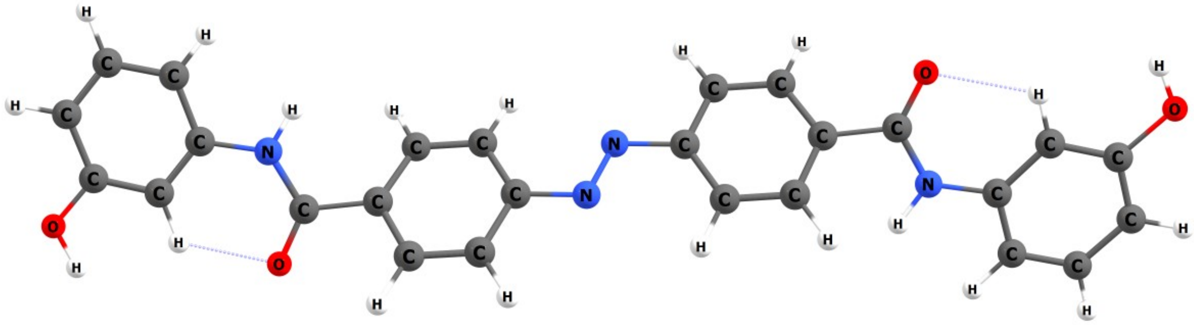

$L1_E$(c)

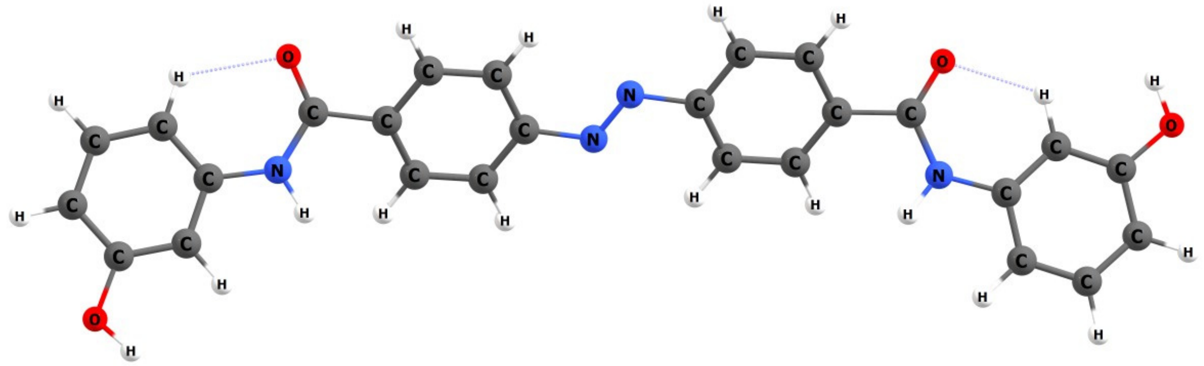

L1$_E$(d)

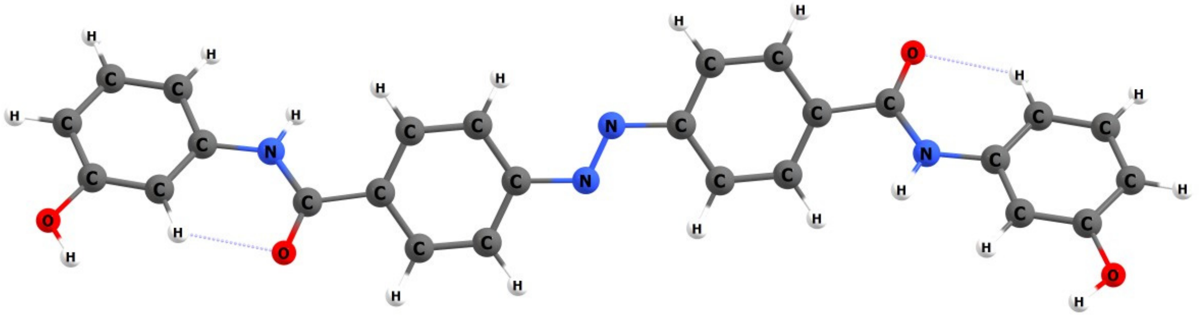

L1$_E$(e)

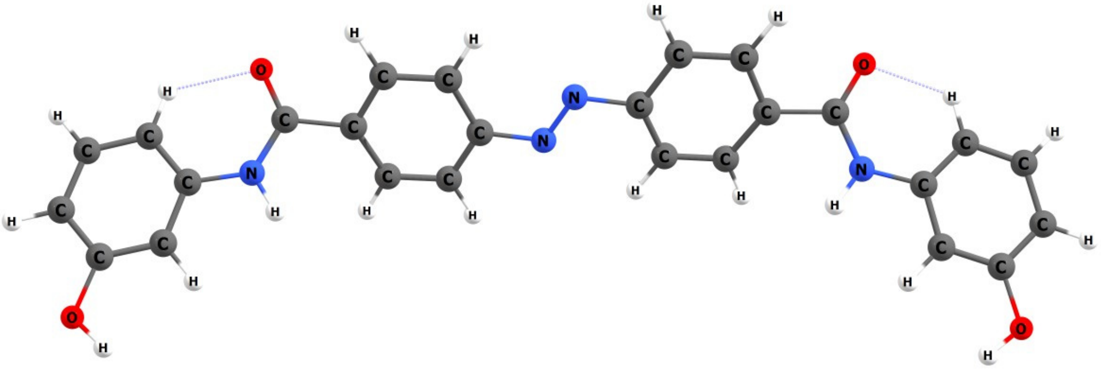

L1$_E$(f)

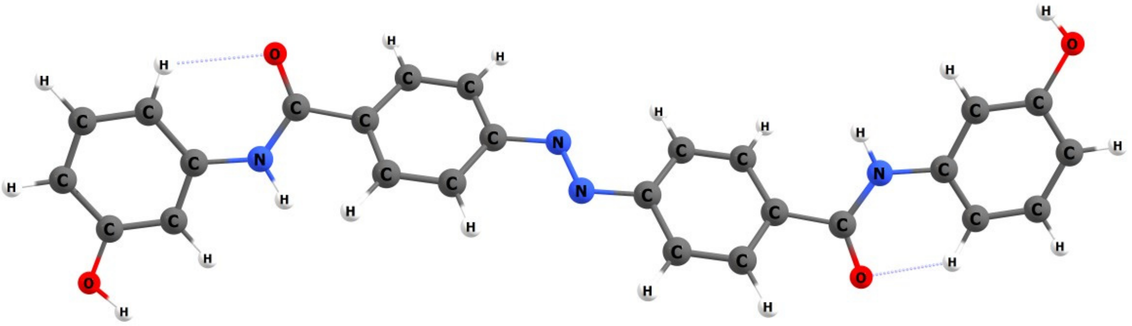

L1$_Z$(a)

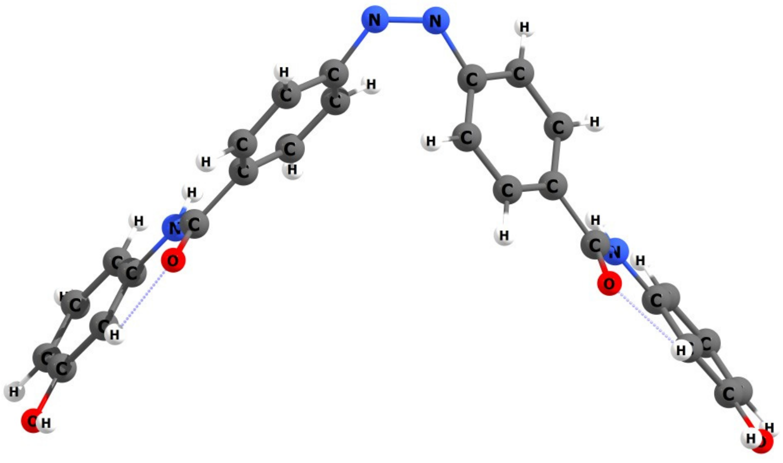

L1$_Z$(b)

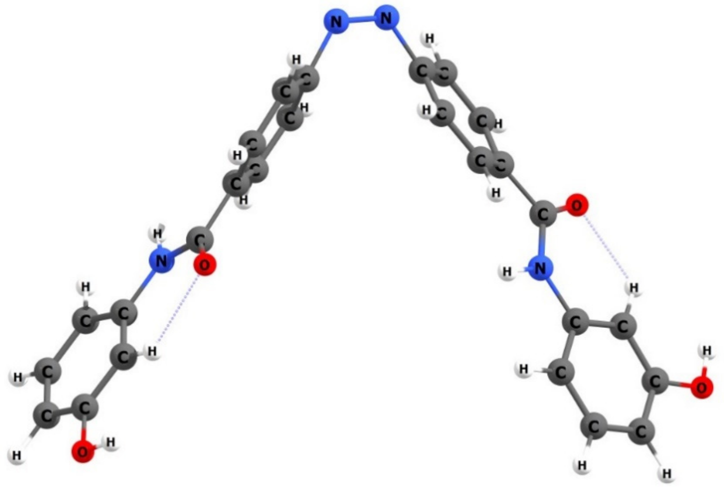

L1$_Z$(c)

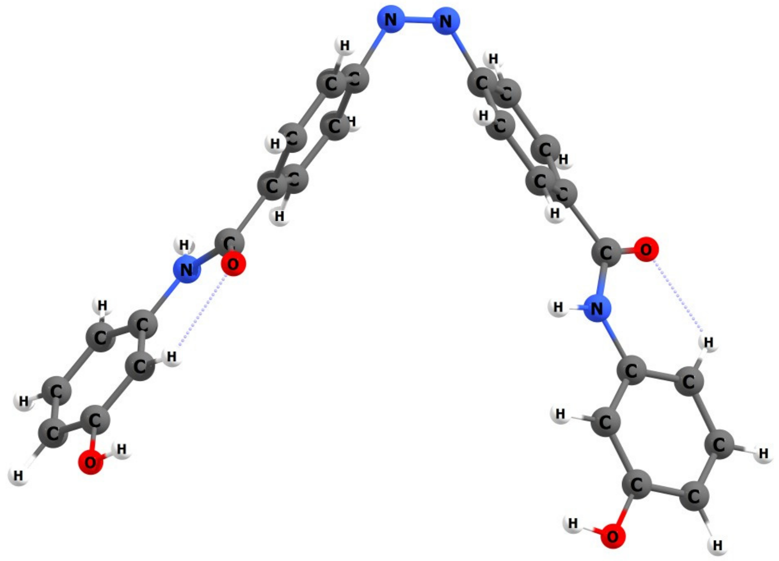

L1$_Z$(d)

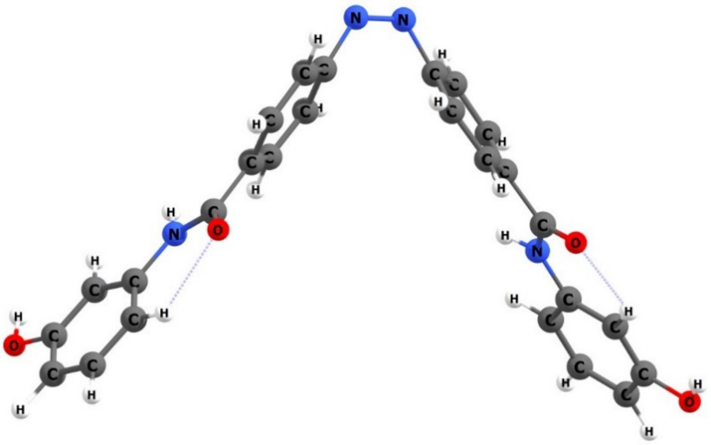

$L1_Z$(e)

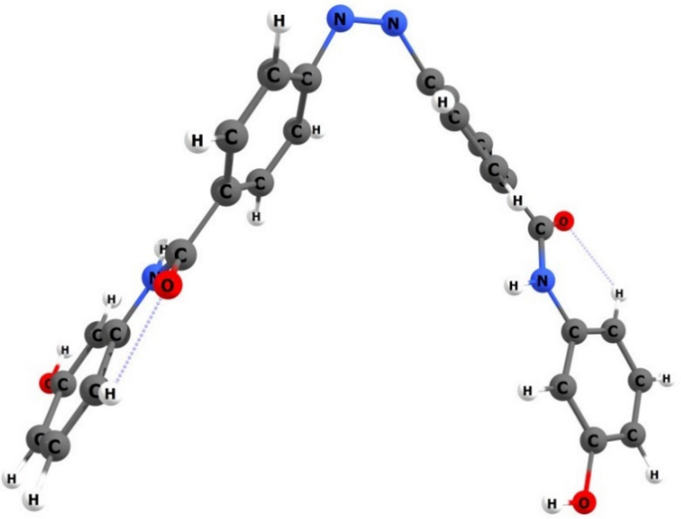

$L1_Z$(f)

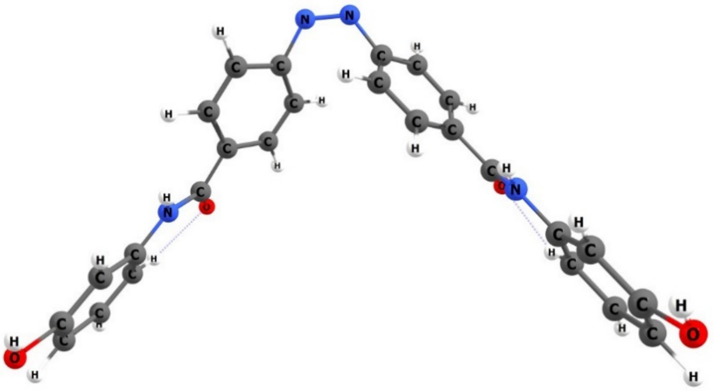

Figure S3. The CAM-B3LYP/6-311++G(d,p) equilibrium structures of $L1_E$ and $L1_Z$ isomers.

Table S2. Relative electronic energies (ΔE in kJ/mol), relative electronic energies with zero-point corrections (ΔE+ZPE in kJ/mol] and Gibbs free energies at T=298.15K (ΔG in kJ/mol) of the systems studied with respect to their most stable isomers. The selected interatomic distances (in Å) and dihedral angles (in degrees) together with dipole moments (in Debye) and Wiberg Bond Indexes (WBI) calculated for the N=N bond are also presented. The results are obtained at the CAM-B3LYP/6-311++G(d,p) level in DMSO.

Species	ΔE	ΔE+ZPE	ΔG	r(CO…CO)	r(N=N)/WBI	∢CNNC	μ
**L2E(a)**	0	0	0	12.001	1.239/1.814	180	0
L2E(b)	0.12	0.61	3.26	12.009	1.239/1.815	179.4	4.3
L2E(c)	5.96	5.53	3.81	12.02	1.239/1.813	179.4	6.36
L2E(d)	6.06	5.67	4.75	12.011	1.239/1.813	179.9	9.03
L2E(e)	11.5	10.78	8.64	12.022	1.239/1.812	180	0
L2E(f)	11.86	10.83	7.13	12.031	1.239/1.812	179.5	14.59
**L2Z(a)**	52.04	52.12	56.52	8.056	1.234/1.950	7.1	5.72
L2Z(b)	52.24	52.81	56.94	8.032	1.234/1.951	6.8	6.99
L2Z(c)	58.14	58.08	60.78	8.182	1.234/1.948	7.1	10.52
L2Z(d)	58.27	58.48	59.99	8.077	1.234/1.948	7.1	6.55
L2Z(e)	64.27	63.76	64.04	8.143	1.234/1.949	7.2	6.18
L2Z(f)	64.59	63.68	63.59	8.134	1.234/1.946	7.2	14.18

L2E(a)

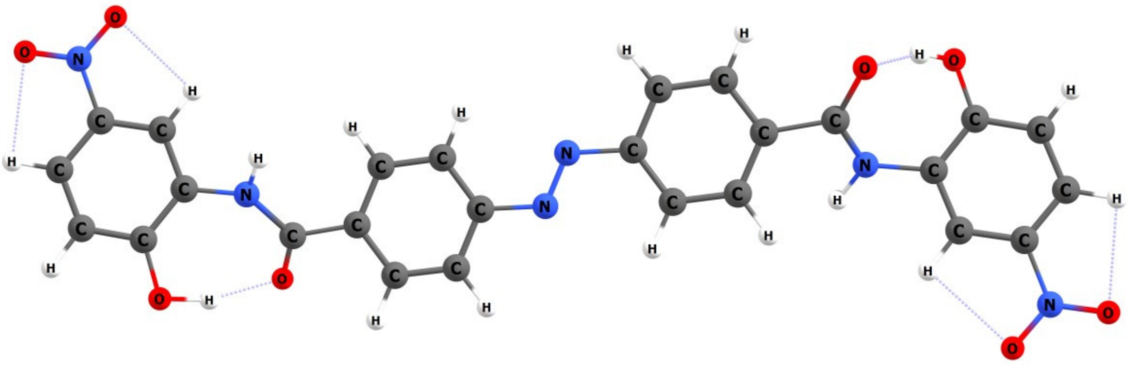

L2E(b)

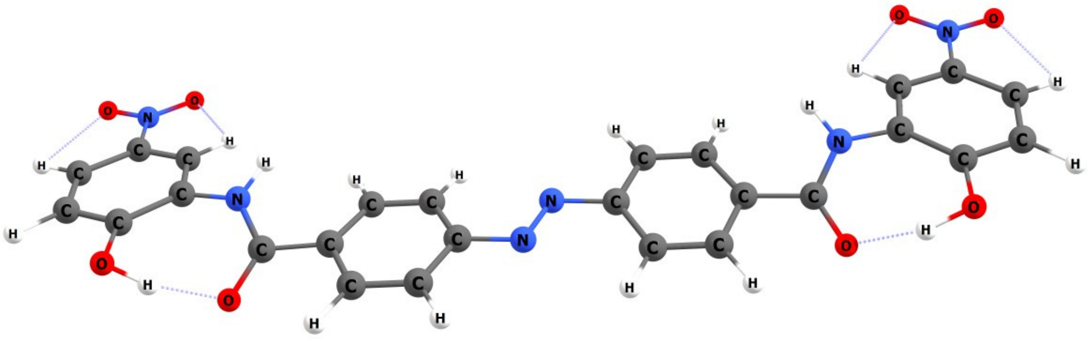

L2E(c)

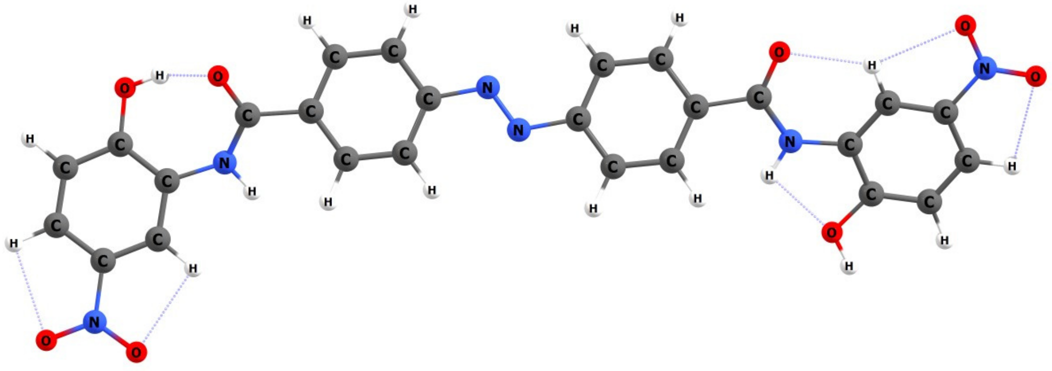

L2E(d)

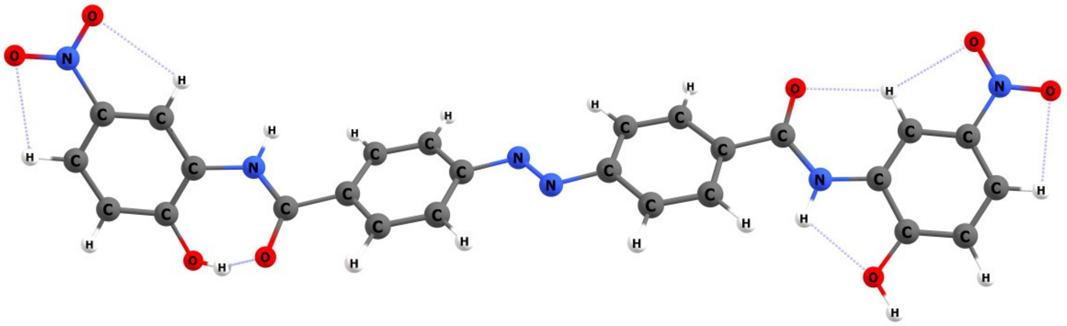

L2E(e)

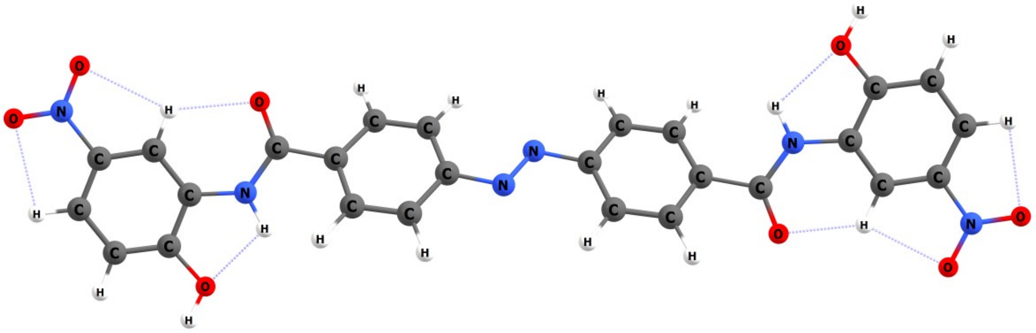

L2E(f)

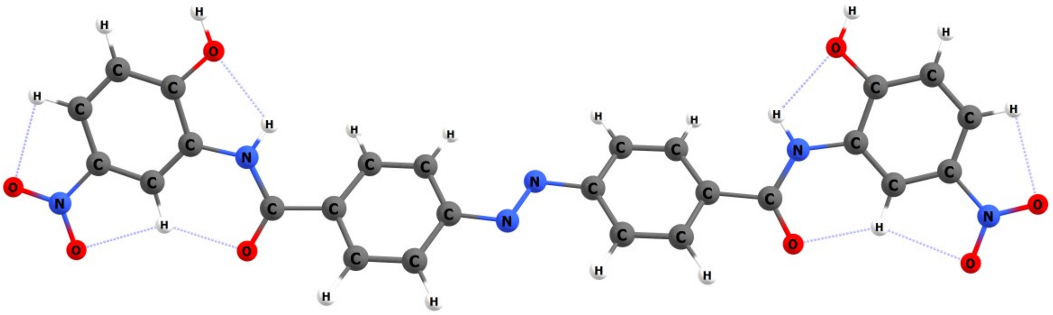

L2Z(a)

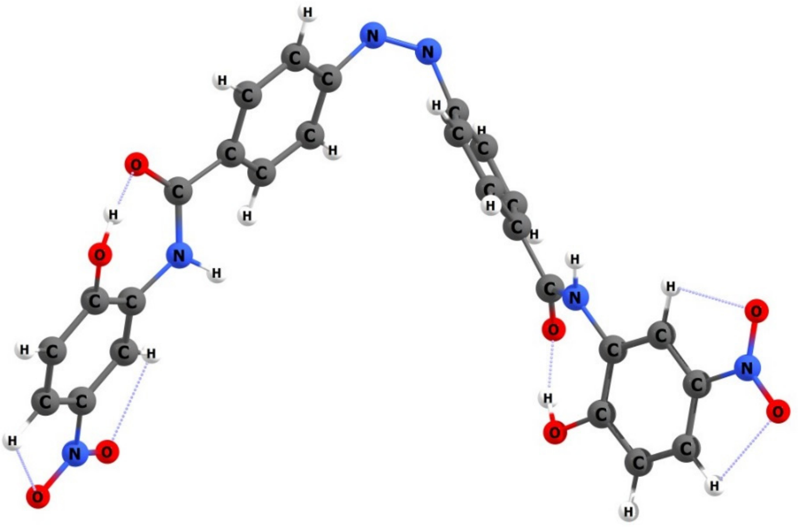

L2z(b)

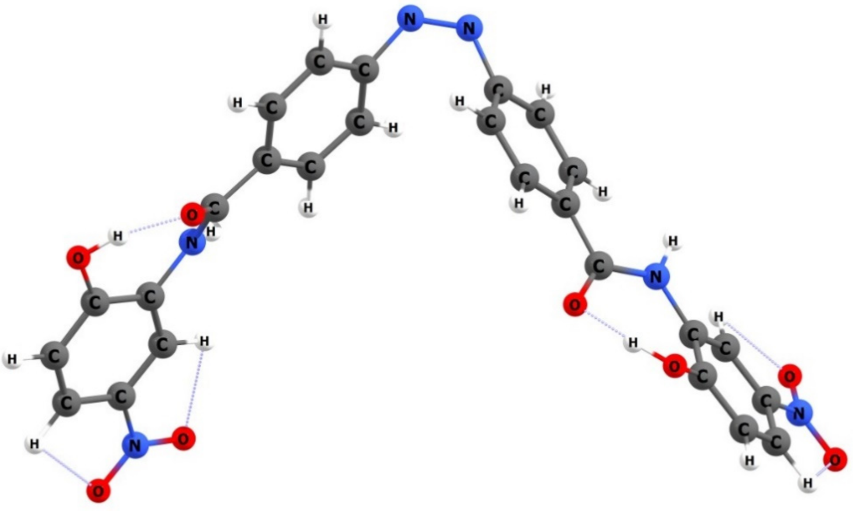

L2z(c)

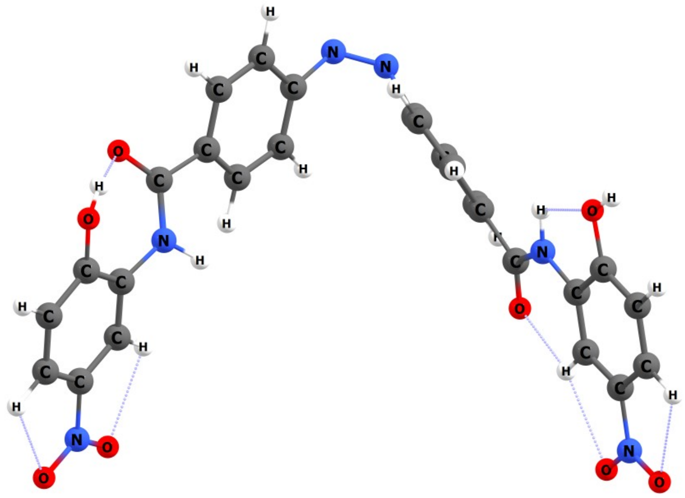

L2z(d)

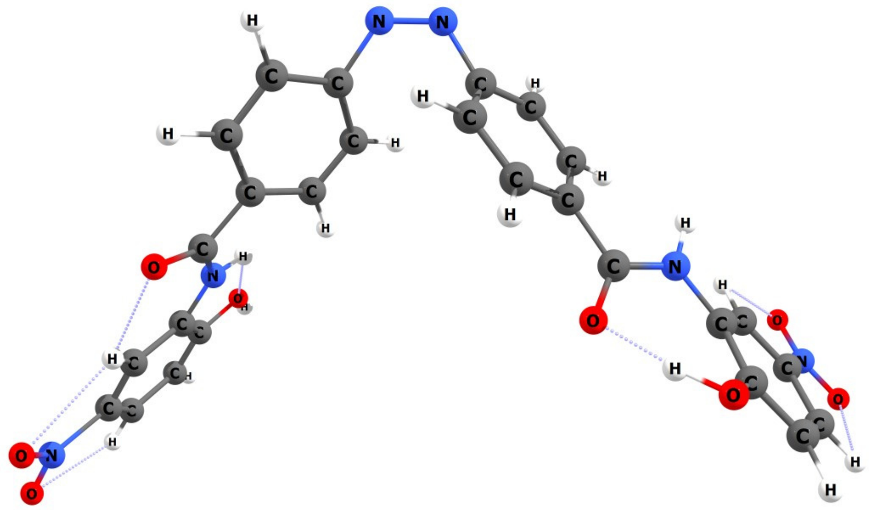

L2Z(e)

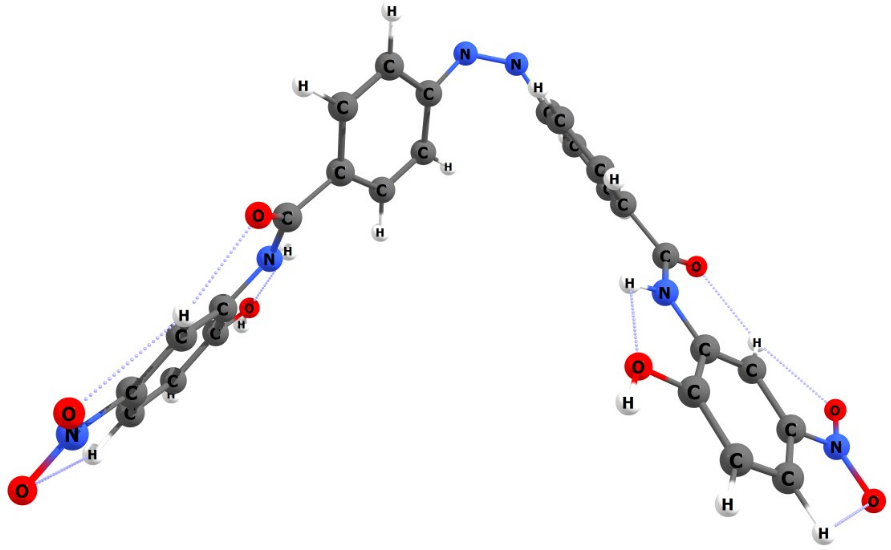

L2Z(f)

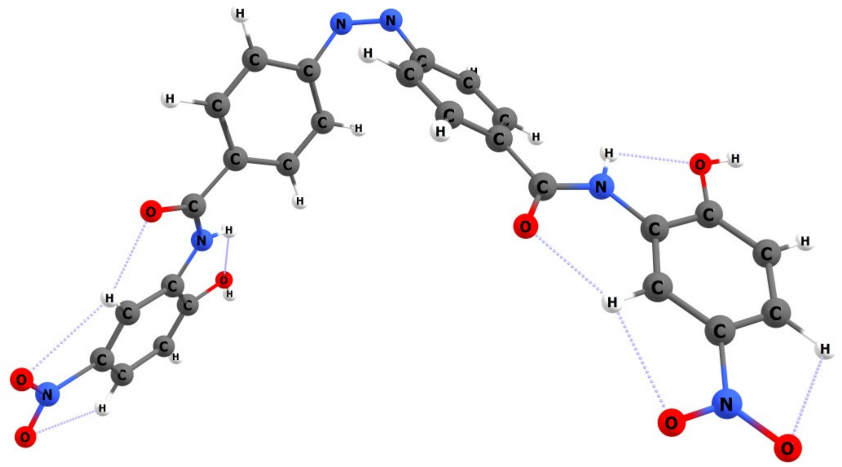

Figure S4. The CAM-B3LYP/6-311++G(d,p) equilibrium structures of L2E and L2Z isomers.

Quantum yield determination

The photon flux of the photoreactor used in UV-irradiation experiments (λ = 365 nm) was estimated by measuring the obtainment of Fe^{2+} ions from potassium ferrioxalate [1].

On the basis of spectrophotometric measurements the rate of ferrous ion formation was determined as: 6.98×10^{-6} M. The mole of photon absorbed per time unit were calculated using the reported quantum yield value of ferrioxalate (1.21) giving the value: 1.36×10^{-8} mol/s.

In order to determine quantum yield of tested amides L1 and L2 the respective solutions (of concentration high enough to absorb all incident light – A_{365} >2) were irradiated in the photoreactor and the absorbance increase at 440 nm and 450 nm for L1 and L2 respectively over time was monitored by UV-Vis spectrophotometry. The molar absorptivities at these wavelengths were used to calculated concentration of *cis*-L1 and *cis*-L2.

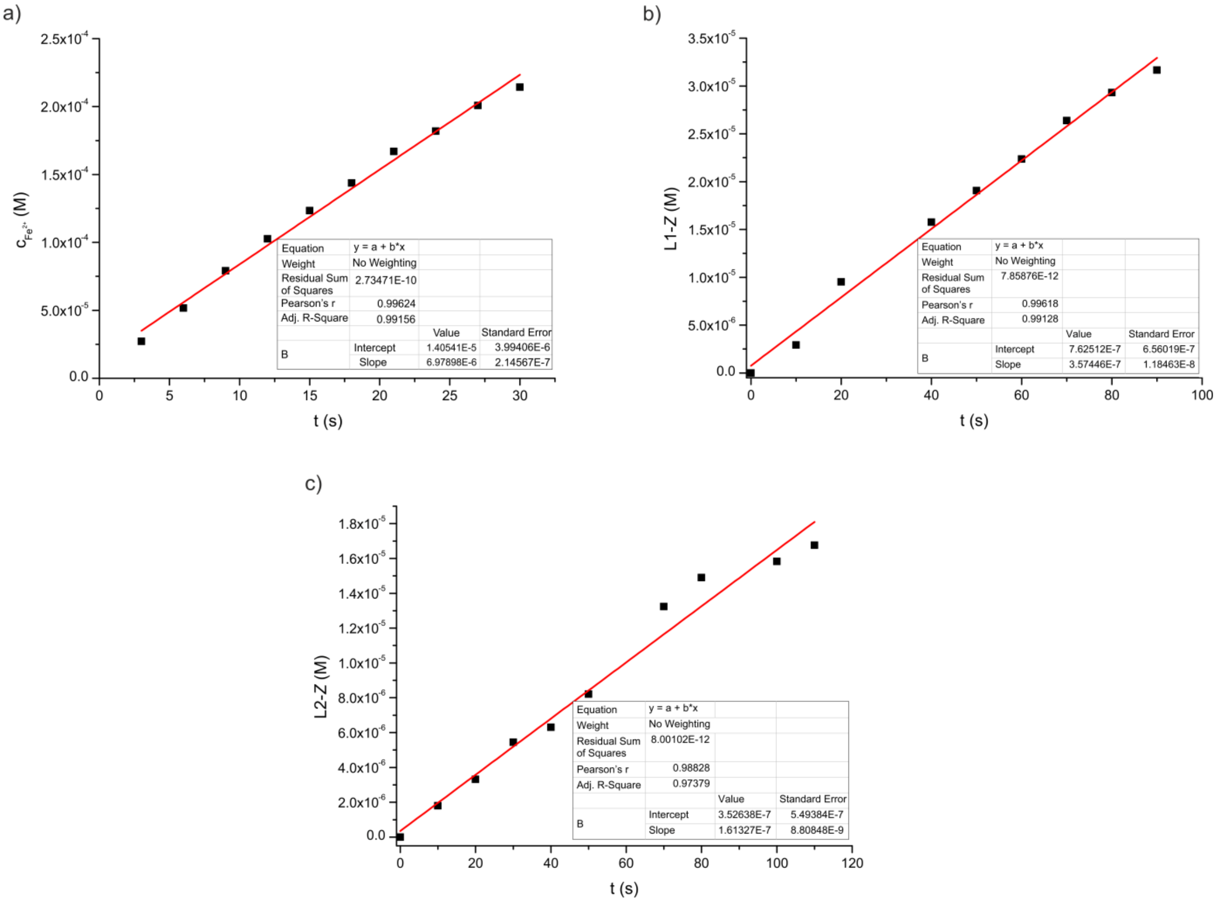

Fig. S5 Concentration of a) Fe^{2+} ions b) *cis*-L1 ($c = 7.33\times10^{-5}$ M) c) *cis*-L2 ($c = 7.62\times10^{-5}$ M) as a function of time (s) during irradiation with UV light (λ =365 nm).

Table S3. Predicted (TD-DFT CAM-B3LYP/6-311++G(d,p) in DMSO) singlet ($S_0\rightarrow S_i$) excitation energies starting from the ground state of L1$_E$(a) and L1$_Z$(a) isomers, with their corresponding oscillator strengths, f.

	**L1$_E$(a)**			**L1$_Z$(a)**	
$S_0\rightarrow S_i$	λ[nm]	f	$S_0\rightarrow S_i$	λ[nm]	F
$^1(n,\pi^*)$	462.42	0.0	$^1(\pi,\pi^*)$	442.68	0.054
$^1(\pi,\pi^*)$	331.17	1.732	$^1(\pi,\pi^*)$	280.94	0.638
$^1(\pi,\pi^*)$	283.0	0.082	$^1(\pi,\pi^*)$	271.48	0.466
$^1(\pi,\pi^*)$	277.37	0.018	$^1(\pi,\pi^*)$	261.9	0.061
$^1(\pi,\pi^*)$	274.56	0.070	$^1(\pi,\pi^*)$	259.63	0.119
$^1(n,\pi^*)$	272.88	0.002	$^1(\pi,\pi^*)$	254.32	0.051
$^1(n,\pi^*)$	257.22	0.005	$^1(\pi,\pi^*)$	252.94	0.017
$^1(n,\pi^*)$	253.93	0.001	$^1(\pi,\pi^*)$	249.34	0.055

$^1(n,\pi^*)$	250.22	>0.001	$^1(n,\pi^*)$	245.95	0.005
$^1(n,\pi^*)$	249.78	0.005	$^1(\pi,\pi^*)$	244.87	0.016
$^1(\pi,\pi^*)$	238.95	0.028	$^1(\pi,\pi^*)$	243.61	0.015
$^1(\pi,\pi^*)$	237.61	0.358	$^1(\pi,\pi^*)$	237.11	0.205
$^1(\pi,\pi^*)$	233.14	0.026	$^1(\pi,\pi^*)$	232.42	0.204
$^1(\pi,\pi^*)$	225.65	0.101	$^1(\pi,\pi^*)$	227.59	0.078
$^1(\pi,\pi^*)$	224.53	0.132	$^1(\pi,\pi^*)$	226.46	0.085

Table S4. Cartesian coordinates (in Å) of the ground state equilibrium structures of $L1_E$(a) and $L1_Z$(a).

**$L1_E$(a)**				**$L1_Z$(a)**			
7	-0.363002000	1.029567000	0.023148000	7	0.635836000	4.787120000	0.514639000
7	0.307410000	-0.011960000	-0.023096000	7	-0.593989000	4.763297000	0.620043000
6	-1.770507000	0.845880000	0.098616000	6	1.419915000	3.619953000	0.231740000
6	1.714127000	0.173988000	-0.104772000	6	-1.380935000	3.601542000	0.323982000
6	-2.401193000	-0.397663000	0.115991000	6	1.374327000	2.493235000	1.044895000
6	-2.524537000	2.013355000	0.162255000	6	2.352717000	3.711275000	-0.795790000
6	-3.780480000	-0.461410000	0.183575000	6	2.250473000	1.446017000	0.808755000
1	-1.807928000	-1.300626000	0.082867000	1	0.665792000	2.433348000	1.860224000
6	-3.904752000	1.943762000	0.242948000	6	3.193362000	2.643940000	-1.051735000
1	-2.014506000	2.968372000	0.149428000	1	2.402056000	4.612034000	-1.394786000
6	-4.543405000	0.707638000	0.241311000	6	3.160604000	1.508302000	-0.244385000
1	-4.255428000	-1.434323000	0.222431000	1	2.186856000	0.568389000	1.440380000
1	-4.498757000	2.846064000	0.302863000	1	3.899739000	2.688665000	-1.870167000
6	-6.039945000	0.696849000	0.342758000	6	4.105671000	0.393189000	-0.576616000
6	2.474451000	-0.988815000	-0.075875000	6	-2.324697000	3.216763000	1.267853000
6	2.337565000	1.417713000	-0.222360000	6	-1.333219000	2.980192000	-0.920965000
6	3.857700000	-0.917710000	-0.142872000	6	-3.168333000	2.151707000	0.999714000
1	1.970817000	-1.944488000	-0.004663000	1	-2.389591000	3.749458000	2.208219000
6	3.713720000	1.481903000	-0.304785000	6	-2.208476000	1.945229000	-1.196387000
				1	-0.628296000	3.311767000	-1.671858000
				6	-3.113198000	1.503800000	-0.233229000
				1	-3.898846000	1.866745000	1.746899000
				1	-2.193321000	1.462870000	-2.164908000
				6	-4.028057000	0.375678000	-0.603209000
				8	-4.359673000	0.188102000	-1.764759000
				7	-4.432543000	-0.405447000	0.436416000
				1	-4.004518000	-0.216091000	1.330160000
				6	-5.315293000	-1.502945000	0.430272000
				6	-5.487844000	-2.182169000	1.638158000
				6	-6.010961000	-1.913314000	-0.704796000
				6	-6.352413000	-3.262334000	1.700444000
				1	-4.948632000	-1.862095000	2.521707000
				6	-6.873517000	-3.000894000	-0.614980000
				1	-5.876019000	-1.393821000	-1.641319000
				6	-7.053065000	-3.684030000	0.580129000
				1	-6.482530000	-3.785232000	2.640090000
				8	-7.575392000	-3.438026000	-1.698997000
				1	-7.730026000	-4.527560000	0.619435000
				8	4.487805000	0.214109000	-1.723975000

1	1.737781000	2.316608000	- 0.252179000
6	4.486036000	0.319295000	- 0.251802000
1	4.430953000	-1.836648000	- 0.141755000
1	4.211495000	2.437192000	- 0.408619000
6	5.974165000	0.467864000	- 0.364368000
8	6.467755000	1.388767000	- 0.998598000
7	6.700395000	-0.477858000	0.292206000
1	6.178174000	-1.122054000	0.866843000
6	8.098466000	-0.640103000	0.349247000
6	8.585431000	-1.640582000	1.192877000
6	8.984150000	0.133485000	- 0.397594000
6	9.950262000	-1.858898000	1.281176000
1	7.897032000	-2.242627000	1.773825000
6	10.349971000	-0.107099000	- 0.291371000
1	8.611441000	0.911862000	- 1.045934000
6	10.846095000	-1.098722000	0.543970000
1	10.322587000	-2.636332000	1.937101000
8	11.253907000	0.623491000	- 1.004066000
1	11.914120000	-1.262036000	0.607332000
8	-6.637456000	1.589116000	0.926478000
7	-6.652356000	-0.355530000	-0.265968000
1	-6.057401000	-0.968146000	-0.803092000
6	-8.023383000	-0.674377000	-0.319174000
6	-8.998040000	0.042543000	0.371385000
6	-8.386996000	-1.773491000	-1.100040000
6	-10.328014000	-0.352744000	0.271796000
1	-8.721175000	0.896202000	0.971286000
6	-9.718534000	-2.145196000	-1.183235000
7	4.498977000	-0.369658000	0.480469000
1	4.199959000	-0.058490000	1.392176000
6	5.344934000	-1.495658000	0.495488000
6	5.849551000	-2.083903000	-0.662108000
6	5.664588000	-2.028933000	1.745914000
6	6.671575000	-3.200635000	-0.551979000
1	5.607724000	-1.671824000	-1.630064000
6	6.483853000	-3.142574000	1.827653000
1	5.272330000	-1.571716000	2.646490000
6	6.995939000	-3.739970000	0.685377000
8	7.190903000	-3.808042000	-1.656620000
1	6.728526000	-3.552009000	2.800174000
1	7.638656000	-4.609056000	0.739900000
1	6.911671000	-3.346647000	-2.454836000
1	-7.373633000	-2.895847000	-2.469262000

1	-7.629362000	-2.332698000	-1.635905000
6	-10.701745000	-1.443000000	-0.502061000
8	-11.316256000	0.317007000	0.930328000
1	-9.995020000	-2.998452000	-1.790515000
1	-11.744661000	-1.726029000	-0.561886000
1	-10.945746000	1.048164000	1.436404000
1	10.801195000	1.276640000	- 1.548575000

HOMO

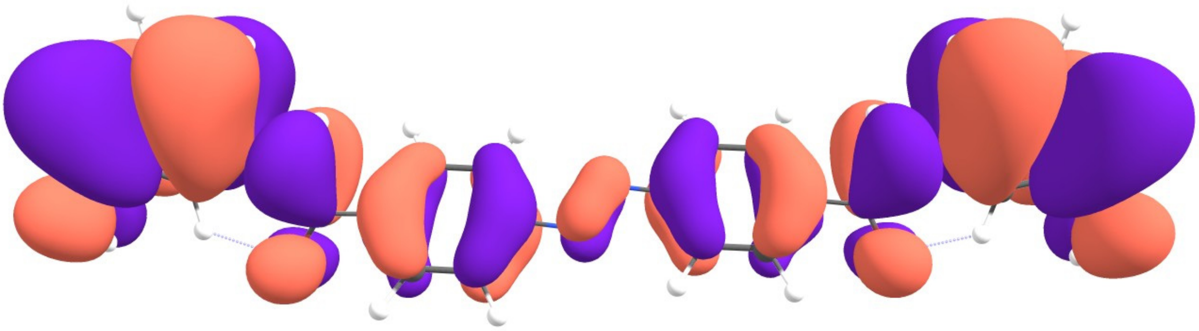

LUMO

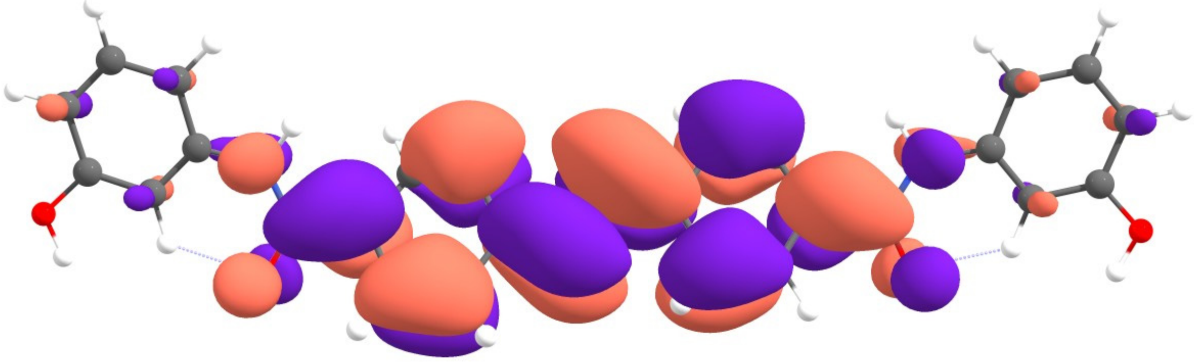

LP (HOMO-5)

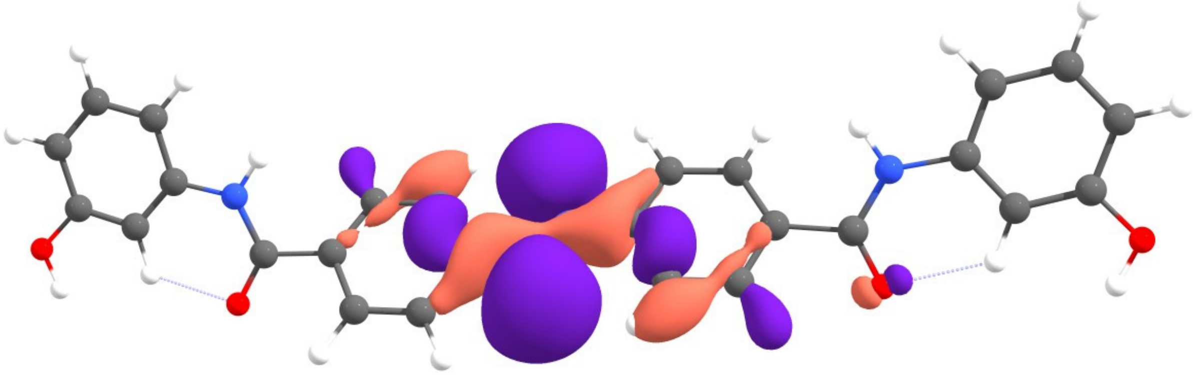

HOMO

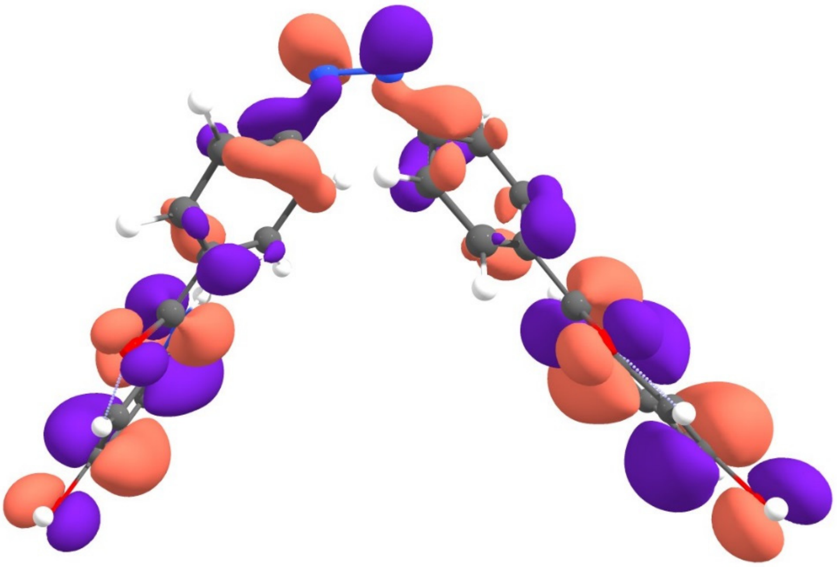

LUMO

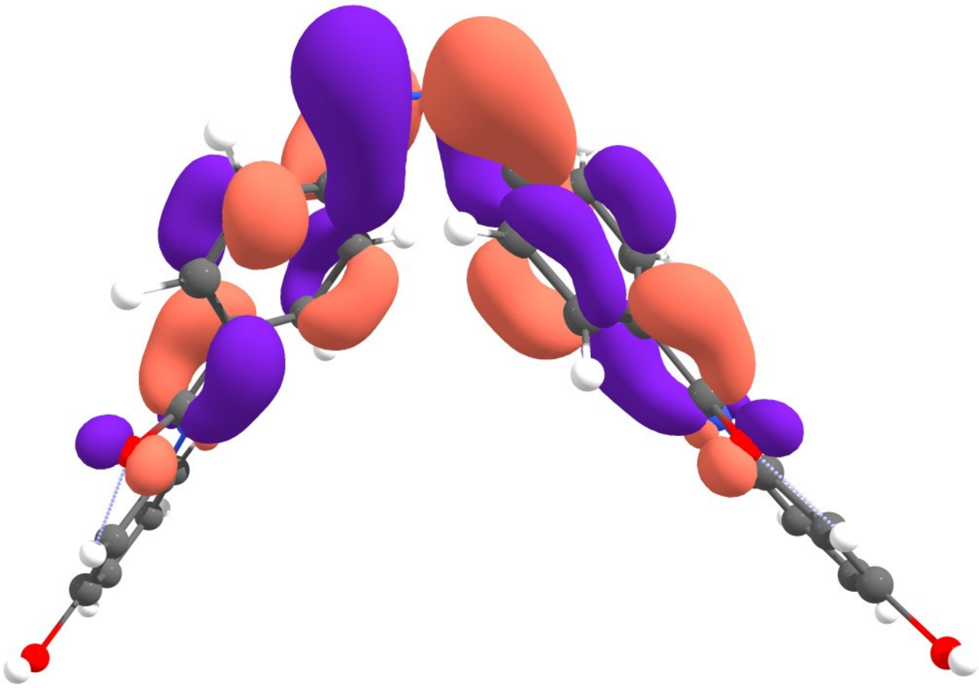

Figure S6. Highest occupied molecular orbitals (HOMO) and lowest unoccupied molecular orbitals.

(LUMO) for the ground singlet state of L1$_E$(a) and L1$_Z$(a). The LP orbital for L1$_E$(a) is also presented.

Table S5. Predicted (TD-DFT CAM-B3LYP/6-311++G(d,p) in DMSO) singlet ($S_0 \rightarrow S_i$) excitation energies starting from the ground Scheme 2. E(a) and L2$_Z$(a) isomers, with their corresponding oscillator strengths, f.

	**L2$_E$(a)**			**L2$_Z$(a)**	
$S_0 \rightarrow S_i$	λ[nm]	f	$S_0 \rightarrow S_i$	λ[nm]	f
$^1(n,\pi^*)$	468.19	0.0	$^1(\pi,\pi^*)$	443.09	0.058
$^1(\pi,\pi^*)$	329.39	1.878	$^1(\pi,\pi^*)$	307.03	0.035
$^1(n,\pi^*)$	306.85	0.0	$^1(n,\pi^*)$	307.02	0.001
$^1(\pi,\pi^*)$	306.83	0.011	$^1(\pi,\pi^*)$	299.67	0.664

$^1(n,\pi^*)$	299.18	0.0	$^1(n,\pi^*)$	299.33	0.002
$^1(\pi,\pi^*)$	298.62	0.489	$^1(\pi,\pi^*)$	278.53	0.445
$^1(\pi,\pi^*)$	276.77	0.116	$^1(\pi,\pi^*)$	275.48	0.012
$^1(n,\pi^*)$	275.64	0.0	$^1(\pi,\pi^*)$	274.54	0.377
$^1(\pi,\pi^*)$	275.42	0.045	$^1(\pi,\pi^*)$	267.86	0.283
$^1(n,\pi^*)$	268.52	0.0	$^1(n,\pi^*)$	264.71	0.001
$^1(n,\pi^*)$	264.82	0.005	$^1(n,\pi^*)$	264.68	0.002
$^1(n,\pi^*)$	264.68	0.0	$^1(\pi,\pi^*)$	260.23	0.234
$^1(\pi,\pi^*)$	261.48	0.189	$^1(\pi,\pi^*)$	256.45	0.120
$^1(n,\pi^*)$	238.52	0.0	$^1(\pi,\pi^*)$	253.10	0.123
$^1(n,\pi^*)$	233.57	0.006	$^1(\pi,\pi^*)$	247.13	0.043

Table S6. Cartesian coordinates (in Å) of the ground state equilibrium structures of $L2_E$(a) and $L2_Z$(a):.

$L2_E$(a)	$L2_Z$(a)
7 0.434665000 0.438734000 0.049162000<br>7 -0.434632000 -0.438907000 -0.048950000<br>6 1.773619000 -0.036615000 0.099852000<br>6 -1.773586000 0.036460000 -0.099682000<br>6 2.752937000 0.946464000 0.175880000<br>6 2.129487000 -1.386284000 0.088554000<br>6 4.091552000 0.591256000 0.225019000<br>1 2.453691000 1.986360000 0.201533000<br>6 3.462718000 -1.736419000 0.151323000<br>1 1.359839000 -2.143262000 0.034618000<br>6 4.453133000 -0.752754000 0.206965000<br>1 4.836757000 1.372054000 0.312081000<br>1 3.755429000 -2.778052000 0.153115000<br>6 5.871490000 -1.205357000 0.286883000<br>6 -2.129442000 1.386120000 -0.088612000<br>6 -2.752918000 -0.946631000 -0.175531000<br>6 -3.462678000 1.736254000 -0.151452000<br>1 -1.359800000 2.143118000 -0.034803000<br>6 -4.091521000 -0.591427000 -0.224747000	7 0.601619000 -4.759746000 0.334115000<br>7 -0.612920000 -4.811350000 0.122116000<br>6 1.323228000 -3.525115000 0.440314000<br>6 -1.401043000 -3.654354000 -0.186757000<br>6 2.474382000 -3.391836000 -0.326249000<br>6 0.983789000 -2.560032000 1.384040000<br>6 3.261573000 -2.262098000 -0.188339000<br>1 2.736992000 -4.163678000 -1.038481000<br>6 1.784942000 -1.442220000 1.528805000<br>1 0.108180000 -2.686607000 2.006768000<br>6 2.926926000 -1.283280000 0.746352000<br>1 4.124618000 -2.149909000 -0.832667000<br>1 1.531954000 -0.682075000 2.255897000<br>6 3.730507000 -0.044379000 0.941592000<br>6 -1.110789000 -2.842392000 -1.279343000<br>6 -2.570064000 -3.469140000 0.541430000<br>6 -1.978272000 -1.818179000 -1.611645000

1 -2.453668000 -1.986532000 -0.200991000<br>6 -4.453098000 0.752594000 -<br>0.206944000<br>1 -3.755359000 2.777895000 -<br>0.153415000<br>1 -4.836734000 -1.372232000 -0.311687000<br>6 -5.871448000 1.205204000 -<br>0.287005000<br>8 -6.155957000 2.292158000 -<br>0.798448000<br>7 -6.801696000 0.380392000<br>0.239853000<br>1 -6.459685000 -0.457113000<br>0.688248000<br>6 -8.218752000 0.463233000<br>0.144115000<br>6 -8.904984000 -0.737107000<br>0.050248000<br>6 -8.945340000 1.667977000<br>0.222709000<br>6 -10.287980000 -0.745424000<br>0.048146000<br>1 -8.363472000 -1.671100000 -0.010267000<br>6 -10.342714000 1.622005000<br>0.209746000<br>8 -8.369917000 2.868114000<br>0.349867000<br>6 -11.023562000 0.429060000<br>0.129615000<br>7 -10.981594000 -2.021731000 -0.045566000<br>1 -10.876366000 2.561033000<br>0.273092000<br>1 -12.103239000 0.399538000<br>0.125556000<br>1 -7.469210000 2.836338000 -<br>0.068770000<br>8 -10.313959000 -3.041735000 -0.109285000<br>8 -12.201735000 -2.014614000 -0.056238000<br>8 6.156036000 -2.292383000<br>0.798154000<br>7 6.801696000 -0.380451000 -<br>0.239902000<br>1 6.459642000 0.457057000 -<br>0.688255000<br>6 8.218771000 -0.463209000 -<br>0.144170000<br>6 8.945440000 -1.667889000 -<br>0.222890000<br>6 8.904907000 0.737177000 -<br>0.050198000	1 -0.223553000 -3.017854000 -<br>1.872962000<br>6 -3.413205000 -2.418017000<br>0.227157000<br>1 -2.810390000 -4.146119000<br>1.351194000<br>6 -3.120582000 -1.581503000 -<br>0.849502000<br>1 -1.772528000 -1.185942000 -<br>2.465010000<br>1 -4.321848000 -2.294700000<br>0.803310000<br>6 -4.006088000 -0.458577000 -<br>1.266287000<br>8 -4.017168000 -0.066806000 -<br>2.437370000<br>7 -4.764473000 0.108533000 -<br>0.303077000<br>1 -4.643059000 -0.245442000<br>0.634432000<br>6 -5.817151000 1.054762000 -<br>0.446025000<br>6 -6.892187000 0.923472000<br>0.418584000<br>6 -5.782688000 2.141825000 -<br>1.342029000<br>6 -7.908698000 1.861338000<br>0.406794000<br>1 -6.935710000 0.095038000<br>1.112132000<br>6 -6.831487000 3.066346000 -<br>1.331460000<br>8 -4.772257000 2.365777000 -<br>2.188467000<br>6 -7.891597000 2.943109000 -<br>0.463014000<br>7 -9.022054000 1.700774000<br>1.330517000<br>1 -6.779353000 3.892579000 -<br>2.028036000<br>1 -8.696370000 3.663472000 -<br>0.455646000<br>1 -4.349567000 1.495621000 -<br>2.416453000<br>8 -9.017236000 0.740524000<br>2.084321000<br>8 -9.912323000 2.535027000<br>1.309814000<br>8 3.196805000 0.994140000<br>1.343576000

6	10.342814000	-1.621812000	- 0.209995000	7	5.052643000	-0.129285000	0.678320000
8	8.370105000	-2.868060000	- 0.350067000	1	5.402812000	-1.039167000	0.416149000
6	10.287902000	0.745601000	- 0.048147000	6	6.012541000	0.918144000	0.600408000
1	8.363316000	1.671115000	0.010432000	6	6.049193000	2.020635000	1.477435000
6	11.023569000	-0.428823000	- 0.129769000	6	7.009356000	0.777053000	- 0.352106000
1	10.876533000	-2.560793000	- 0.273464000	6	7.094448000	2.942873000	1.368334000
7	10.981421000	2.021945000	0.045693000	8	5.151395000	2.219980000	2.447758000
1	12.103243000	-0.399212000	- 0.125764000	6	8.033019000	1.704291000	- 0.425666000
1	7.469369000	-2.836335000	0.068527000	1	6.998414000	-0.062977000	- 1.033007000
8	10.313715000	3.041895000	0.109493000	6	8.091041000	2.794777000	0.431394000
8	12.201564000	2.014917000	0.056363000	1	7.099474000	3.779213000	2.054685000
				7	9.070286000	1.526062000	- 1.431113000
				1	8.899225000	3.507306000	0.355786000
				1	4.289989000	1.805053000	2.175532000
				8	8.997302000	0.561592000	- 2.175992000
				8	9.968291000	2.350546000	- 1.484271000

HOMO

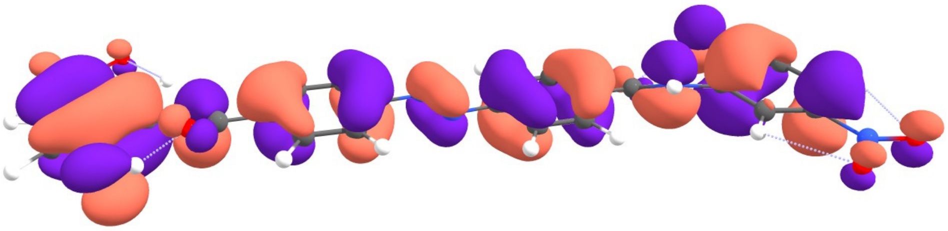

LUMO

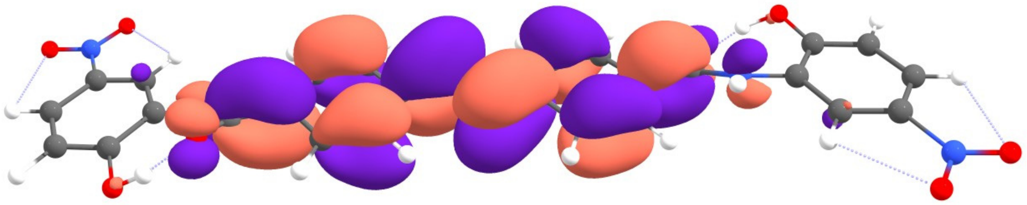

LP (HOMO-3)

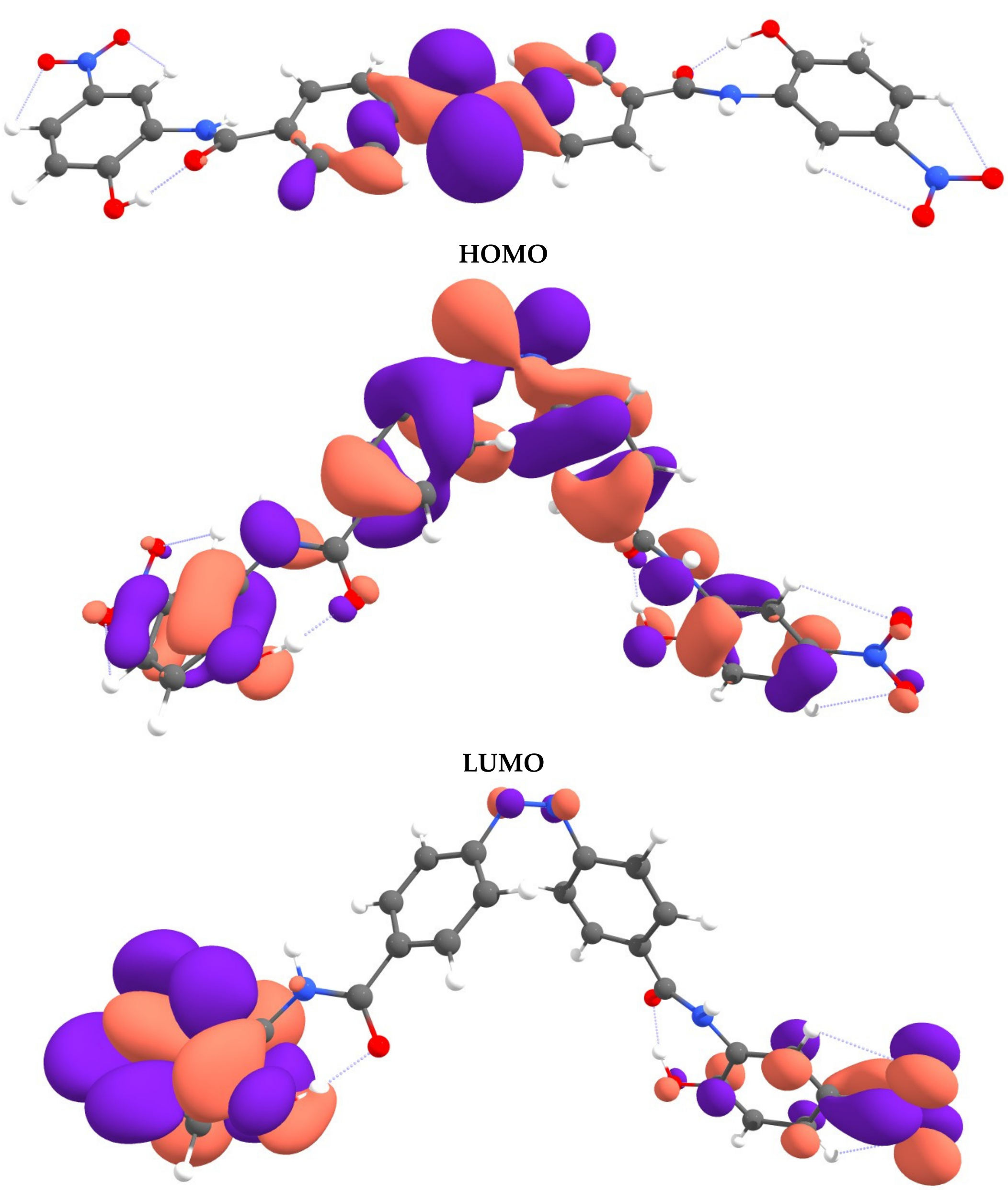

Figure S7. Highest occupied molecular orbitals (HOMO) and lowest unoccupied molecular orbitals.

(LUMO) for the ground singlet state of L2$_E$(a) and L2$_Z$(a). The LP orbital for L2$_E$(a) is also presented.

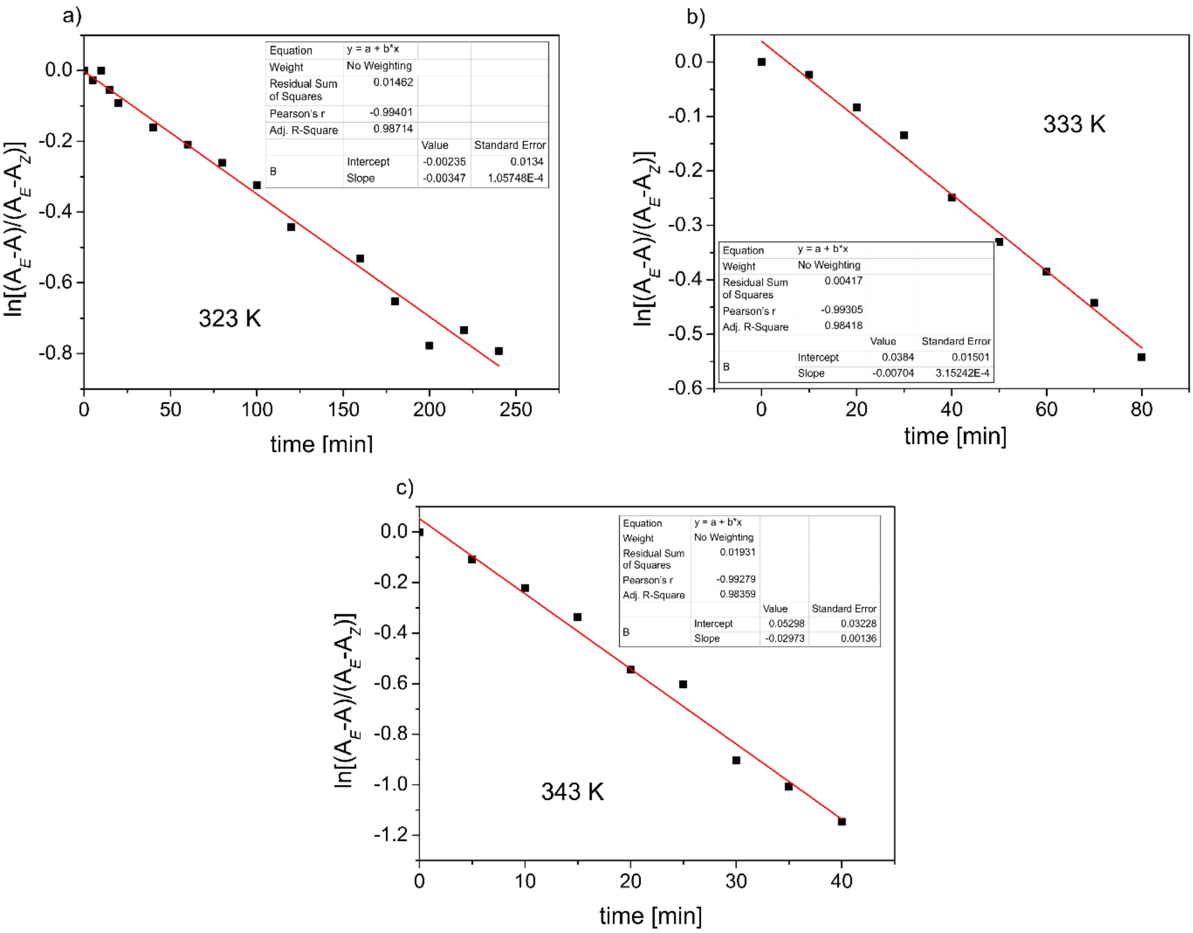

Figure S8. The first order reaction plot obtained for *cis*-enriched L1 solution in DMSO (3.24×10^{-5} M) at a) 323K b) 333K and c) 343K in darkness.

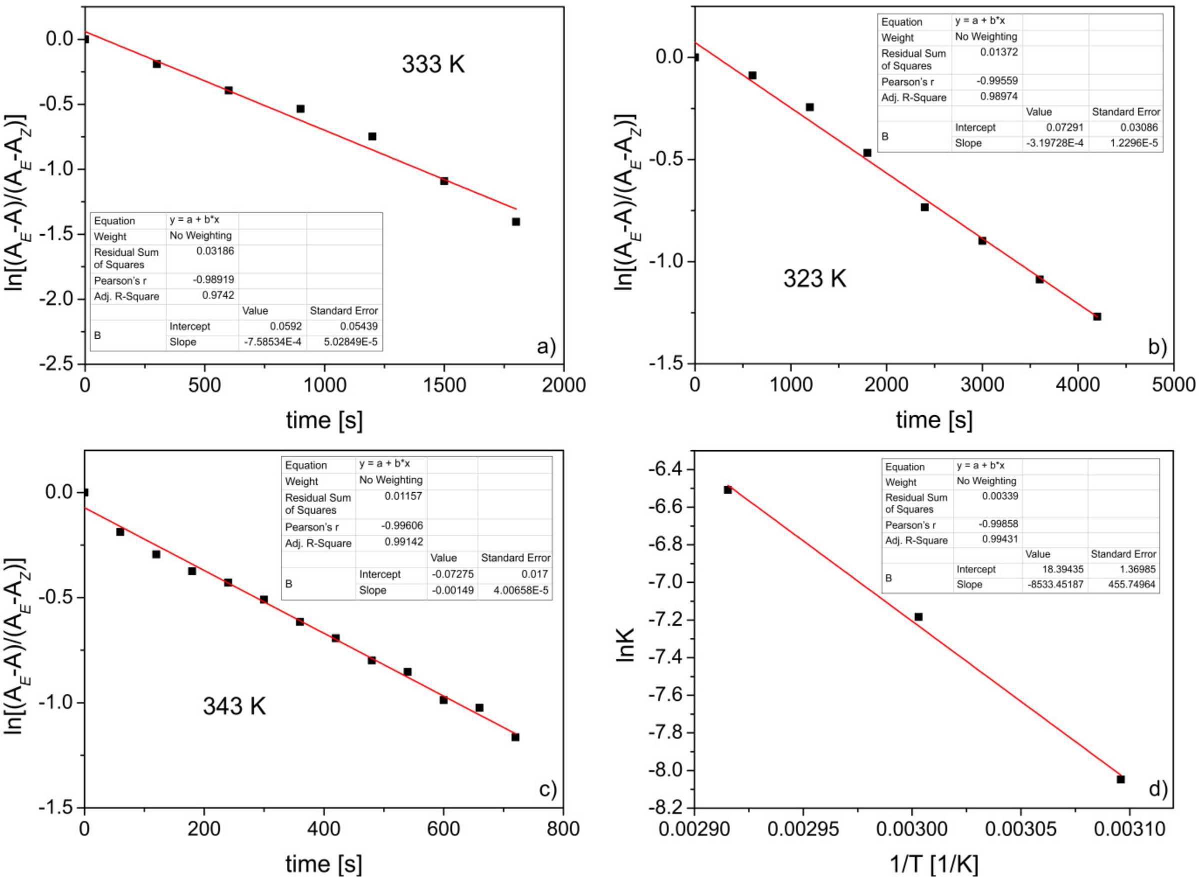

Figure S9. The first order reaction plot obtained for *cis*-enriched L2 solution in DMSO (2.05×10^{-5} M) at a) 333.15K b) 323.15K c) 343.15K in darkness; d) the Arrhenius plot obtained for L2 solution in DMSO.

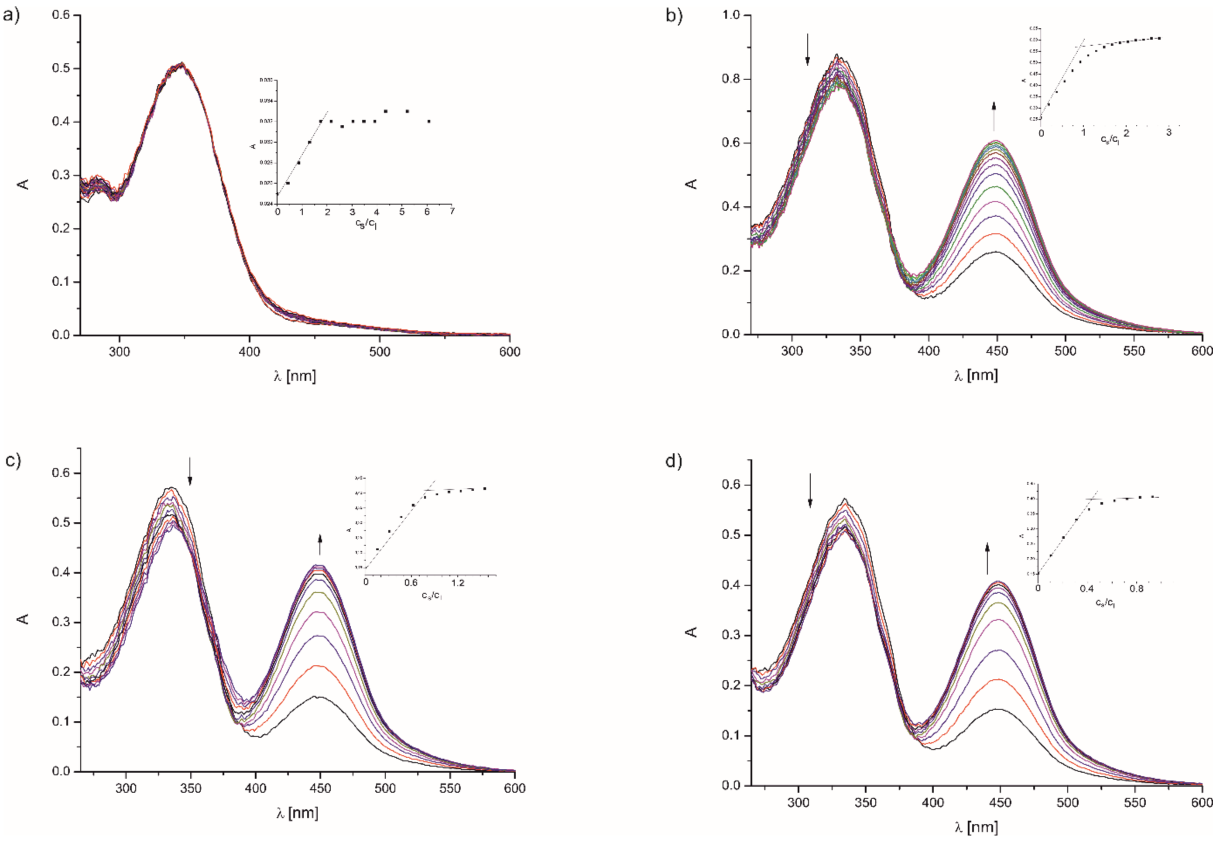

Fig. S10 Spectral changes registered upon a) L1 solution (c = 3.23×10^{-5} M) titration with tetra-*n*-butylammonium fluoride (c = 0-6.57×10^{-4} M), b) L2 solution (c = 2.04×10^{-5} M) titration with tetra-*n*-butylammonium fluoride (c = 0-4.36×10^{-5} M), c) L2 solution (c = 1.62×10^{-5} M) titration with tetra-*n*-butylammonium acetate (c = 0-3.11×10^{-5} M), d) L2 solution (c = 1.62×10^{-5} M) titration with tetra-*n*-butylammonium dihydrogen phosphate (c = 0-1.87×10^{-5} M) in DMSO.

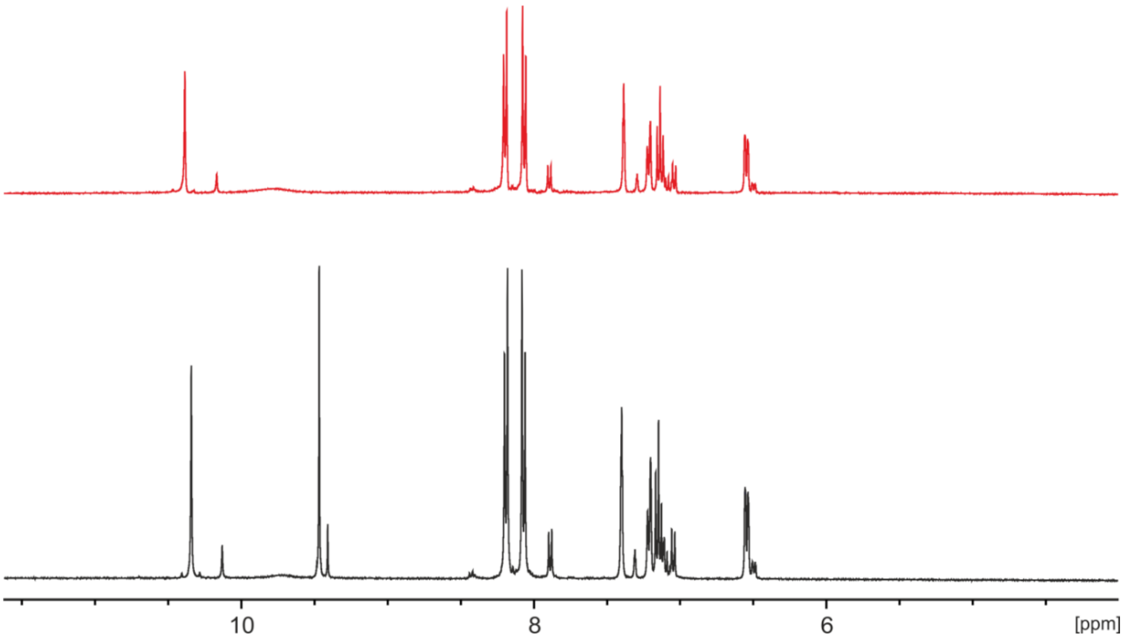

Figure S11. ^{1}H NMR spectra of *cis*-enriched mixture of L1 (c = 17.7 mM, bottom, black) and in the presence of equimolar amount of tetra-*n*-butylammonium fluoride (top, red) registered in DMSO-d_6.

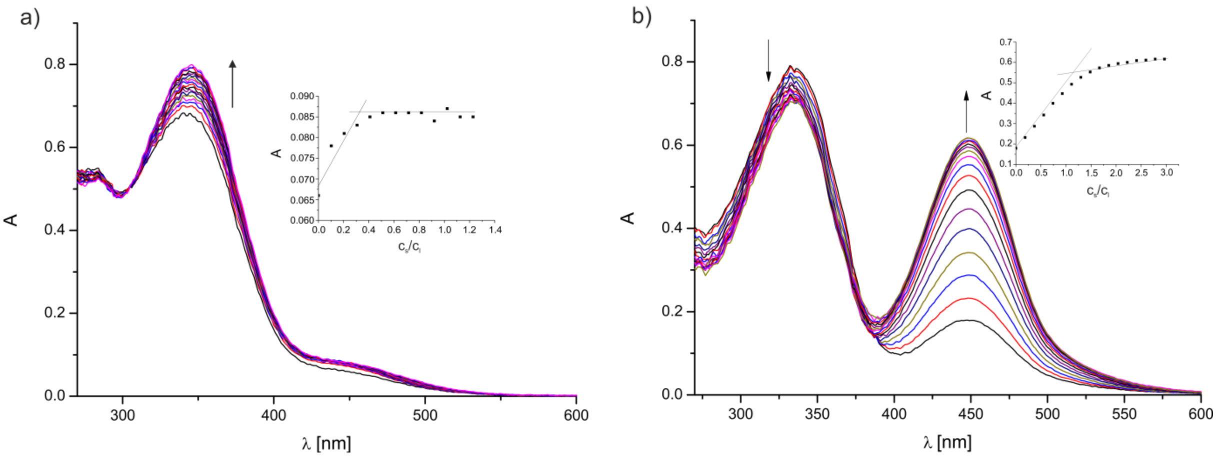

Figure S12. Spectral changes registered upon titration of *cis*-enriched mixture of a) L1 solution (c = 6.18×10^{-5} M) with tetra-*n*-buTable 0. 04×10^{-4} M) b) L2 solution (c = 1.62×10^{-5} M) with tetra-*n*-butylammonium fluoride (c = 0-4.64×10^{-4} M) after UV-light irradiation (λ = 365 nm).

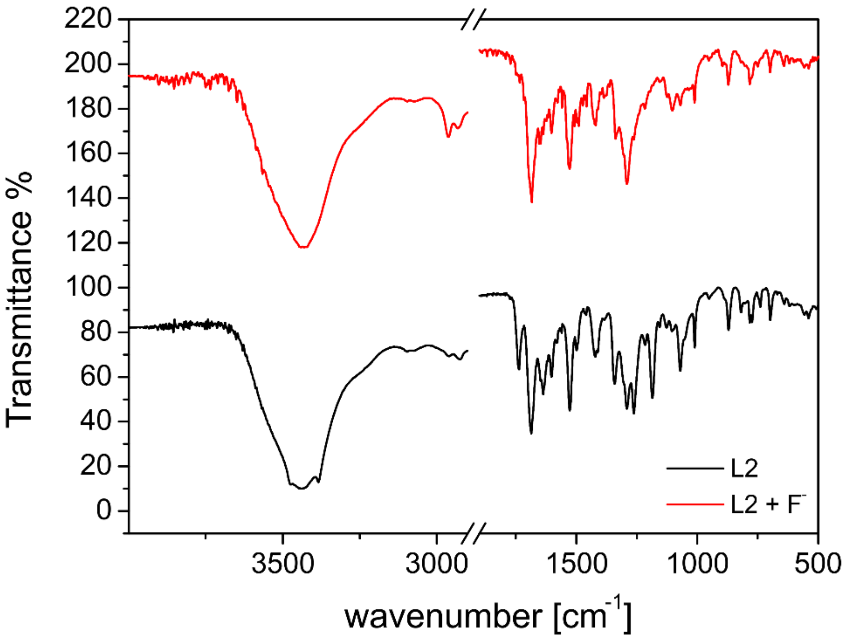

Figure S13. Comparison of FTIR spectra (KBr pellet) of L2 and its mixture with equimolar amount of tetra-*n*-butylammonium fluoride. .

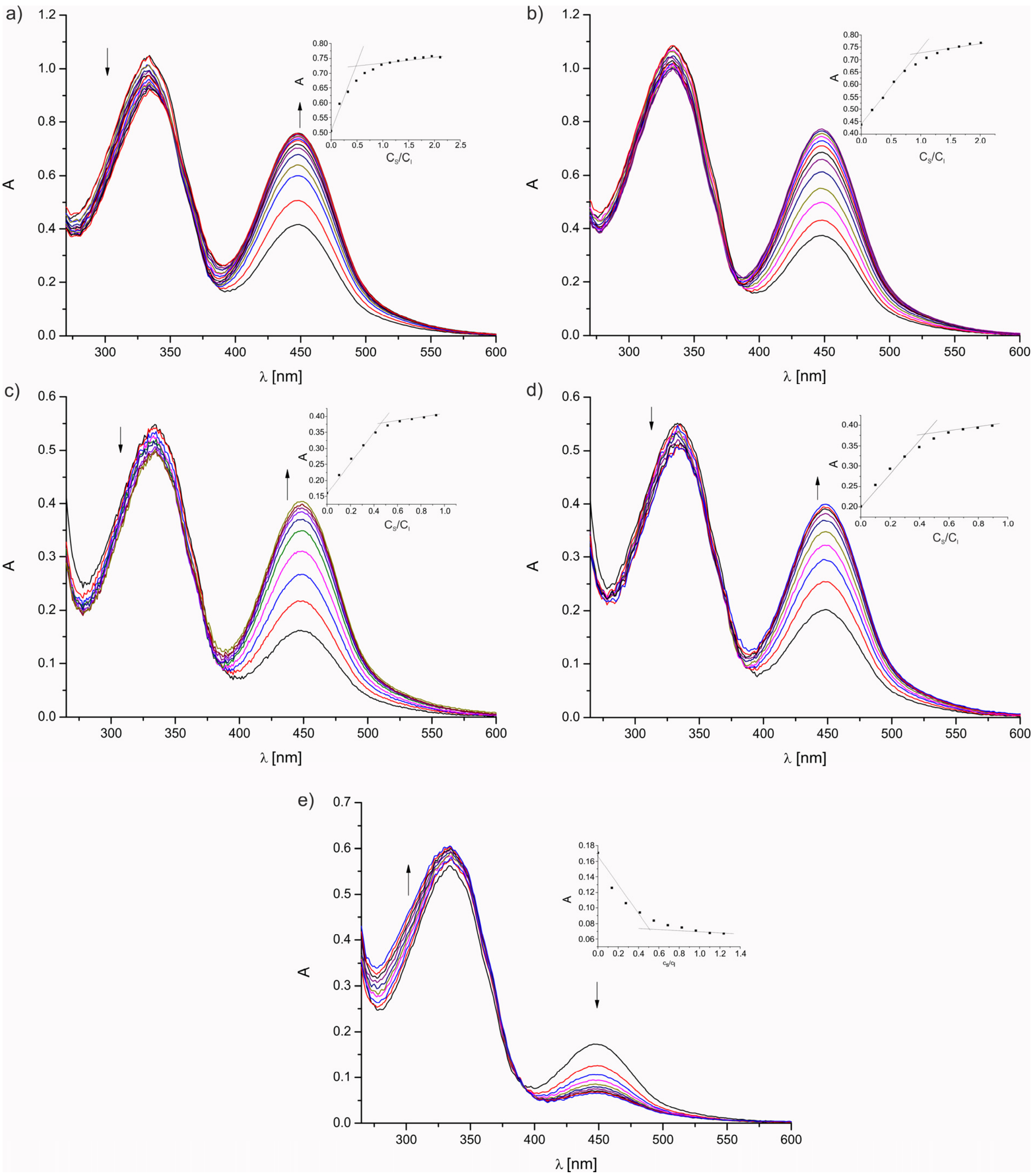

Figure S14. Spectral changes registered upon titration of L2 solution a) (c = 2.41×10^{-5} M) with tetra-*n*-butylammonium fluoride (c = 0-3.33×10^{-5} M), b) (c = 2.41×10^{-5} M) with tetra-*n*-butylammonium acetate (c = 0-3.18×10^{-5} M), c) (c = 1.62×10^{-5} M) with tetra-*n*-butylammonium dihydrogen phosphate (c = 0-1.87×10^{-5} M), d) (c = 1.62×10^{-5} M) with tetra-*n*-butylammonium benzoate (c = 0-1.79×10^{-5} M), e) (c = 1.70×10^{-5} M) with copper(II) perchlorate (c = 0-2.49×10^{-5} M) in DMSO at 333.15K.

References

1. Heath, H. A new sensitive chemical actinometer - II. Potassium ferrioxalate as a standard chemical actinometer. *Proc. R. Soc. London. Ser. A. Math. Phys. Sci.* **1956**, *235*, 518–536, doi:10.1098/rspa.1956.0102.
